# Double-Sided Pressure-Sensitive Adhesive Materials under Human-Centric Extreme Environments

**DOI:** 10.1021/acsami.4c09327

**Published:** 2024-09-02

**Authors:** Jisoo Jeon, Jinyoung Kim, Sehyun Park, Gwendolyn Bryan, Timothy J. Broderick, Morley Stone, Vladimir V. Tsukruk

**Affiliations:** † School of Materials Science and Engineering, Georgia Institute of Technology, Atlanta, Georgia 30332, United States; ‡ Institute for Human and Machine Cognition, Pensacola, Florida 32502, United States; § Department of Intelligent Systems and Robotics, University of West Florida, Pensacola, Florida 32514, United States

**Keywords:** pressure-sensitive adhesive, lap shear test, human-centric extreme environment, long-term adhesion stability, wearable sensors

## Abstract

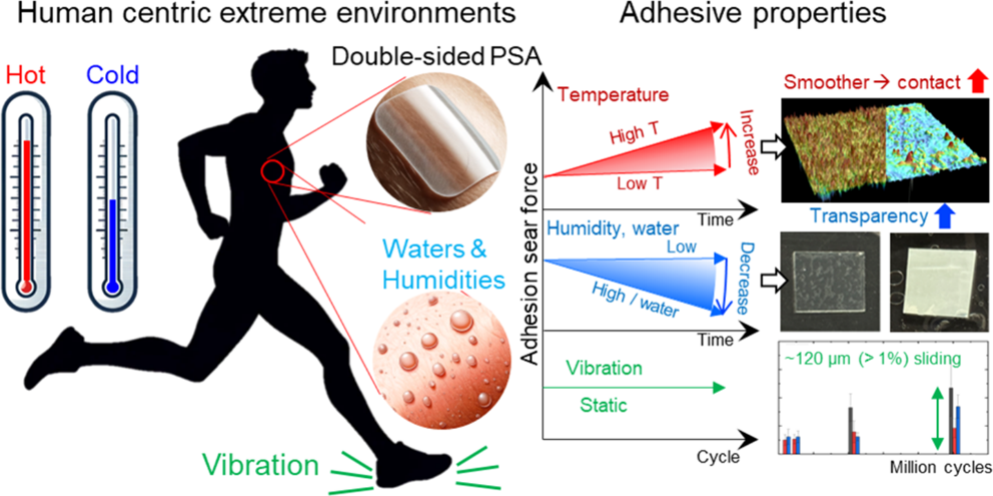

Maintaining the adhesion strength of flexible pressure-sensitive adhesives (PSAs) is crucial for advanced applications, such as health monitoring. Sustainable mounting is critical for wearable sensor devices, especially under challenging surroundings such as low and high temperatures (e.g., polar regions or deserts), underwater and sweat environments (physical activity), and cyclical shear complex stresses. In this article, we consider the adhesive, mechanical, and optical properties of medical-grade double-sided PSAs by simulating extreme human-centric environments. Diverse temperature conditions, water and humidity exposures, and cyclical loads were selected and tested over long intervals, up to 28 days. We observed that high temperatures increased the shear adhesion strength due to the pore closing and expanding contact area between the adhesive layer and substrate. Conversely, low temperatures caused the adhesive layers to harden and reduce the adhesive strength. Immersion in salty and weakly acidic water and excessive humidity reduced adhesion as water interfered with the interfacial interactions. PSA films showed either adhesive or cohesive failure under extreme mechanical stresses and cyclical loading, which is also affected by the presence of various polar solvents. We demonstrated that the variable adhesive performance, mechanical properties, and optical transparency of pressure-sensitive materials can be directly related to changes in their morphologies, surface roughness, swelling state, and alternation of the mechanical contact area, helping to establish the broader rules of design for wearable human health monitoring sensors for the long-term application of wearable devices, sensors, and electrodes.

## Introduction

Pressure-sensitive adhesives (PSAs) are versatile soft polymeric materials that rapidly bond diverse objects under low compressive pressure but can be easily released under modest shear stresses.^1−3^ This behavior is in striking contrast to the regular structural adhesives based upon rigid epoxy and cyanoacrylate materials, which are chemically cross-linkable and exhibit a higher and permanent adhesion strength. Moreover, their applications require the mixing of different precursors or exposure to specific conditions such as temperature, humidity, and light for curing and cannot be released without damaging the substrates.^4,5^

The adhesion in PSA materials, which are mostly composed of acrylate derivatives, occurs through weak mechanical bonds facilitated by the interlocking of small pores and asperities on the surfaces through multiple van der Waals forces.^6^ Their quick and simple dynamic bonding and debonding are widely used in various applications, including packaging, construction, electronics, medicine, and everyday life, to adhere various objects to diverse substrates.^7−11^ In particular, PSA tapes are widely utilized in various applications for wearable sensor mounting for human state and health monitoring during every day activities by bonding various sensors to human skin.^12−14^ Maintaining the adhesion strength of dry medical graded PSAs under human-centric extreme environments is crucial to keep reliable health monitoring with wearable sensors under variable surroundings.

The popular PSA materials are mostly composed of soft polymer-based components such as acrylics,^15^ silicones,^16^ or polyurethanes^17^ made into supported tapes, thin films, or foams.^6^ Among them, acrylic PSAs are most widely used because the low glass transition temperature (*T*_g_) facilitates an expanded conformal mechanical contact under low pressure that generates high interfacial strength, important for applications in wearable device holding. In addition, the high transparency of acrylic materials enables these tapes to be applied for optical sensors without significant interferences and optical losses.^18,19^ Some recent studies demonstrated their retention of high and long-lasting adhesive performance under high humidity and underwater conditions,^20−22^ high temperature,^10,23^ or mechanical stresses.^24^ However, the long-term stability of the adhesion strength of PSAs under relatively harsh environmental conditions has been rarely addressed.

Herein, we consider dry double-sided PSAs for long-term adhesive performance as well as changes in adhesive performance under challenging human-centric conditions, including underwater conditions, high/low humidity, high/low temperature, and cyclical shearing loading. For this task, we selected five commercial medical-grade double-sided PSAs, which are widely used for wearable applications. We characterized their general appearance, microstructure, morphologies, adhesive strength, and mechanical properties, as well as optical transmittance in a wide spectral range before/after diverse treatments. Long-term and stable adhesion is crucial to maintaining continuous human-sensor-machine interfaces and performance in terms of reproducible monitoring during outdoor activity missions.

Furthermore, we depicted three PSAs with higher adhesive performance and evaluated their long-term adhesive performance under different human-centric environmental conditions. We tested the adhesive behavior of these PSAs for up to 28 days and suggested how morphology and property changes control the long-term adhesive performance of different PSA materials under diverse external conditions. The other property changes, including mechanical, optical, chemical, and morphologies, were also evaluated to reveal the mechanism of changes in adhesion properties. These evaluations under different environmental conditions provide further insights into the importance of malleable adhesion performance at the human-machine interface for human and robot state monitoring with multifunctional electrical, electrochemical, and optical wearable sensors.

## Experimental Section

### Materials

Five different popular commercial medical-graded, double-sided PSAs available for the sensor research community were chosen: PSA 1 (type 9889) was provided by 3 M and four other commercial PSAs, were purchased from different commercial sources (see the materials data sheets summarized in Figures S1–S5). Overall, the PSAs studied here are designated as 3M, Secretape, PRO1502, VAPON, and Masktite according to the manufacturer data sheets (see Supporting Information).

The moisture-resistant commercial polyester film was purchased from McMaster-Carr and used as a model substrate representing common plastic sensor boxes. Synthetic seawater prototype (8363–1) was purchased from RICCA. Artificial perspiration (sweat prototype) (1700–0020) was purchased from Pickering Laboratories.

### UV–Vis-NIR Spectroscopy

The optical transmittance of each PSA film was characterized by a Cary 5000 ultraviolet–visible (UV–vis)/NIR spectrometer. The range of wavelength was from 300 to 1600 nm, which covers the visible, NIR, and IR spectral ranges common for optical sensors.

### Fourier Transform Infrared Spectroscopy (FT-IR)

To evaluate the chemical structure of the adhesives, FT-IR spectroscopy (Nicolet 6700, Thermo Fisher Scientific) was conducted in an attenuated total reflection (ATR) mode. The spectral range was between 400 and 4000 cm^–1^.

### Optical Microscopy (OM)

Optical microscopy images were obtained with an Olympus BX51 microscope. The images were taken in reflection mode under bright field conditions to characterize the appearance and uniformity of the samples.

### Scanning Electron Microscopy (SEM)

The surface and cross-sectional SEM images of each adhesive tape were obtained with a Hitachi S-3400 microscope. PSAs were sputtered with gold before measurements. The PSAs were flipped under liquid nitrogen to capture the interfacial morphologies between the substrate and PSA.

### 3D Optical Surface Profile

The 3D surface profiles of PSAs were obtained by a Keyence VK-X3000 optical profilometer. The surface roughness (*S*_a_) was calculated from the surface profiles.

### Contact Angle Measurements

Contact angle was measured by an optical contact angle meter (KSV CAM 101) with a CCD camera (DMK 23U618, Imaging Source) attached to a uniaxial linear stage with *z*-axis stage. Twenty μL of a water droplet was deposited on the PSAs by a micropipette. The contact angles were measured by ImageJ software at multiple locations. The contact angles on PSA films that were treated with salty water were measured after quick removal of liquid with adsorbing paper.

### Tensile Test

The tensile test was conducted by a universal tensile machine (EZ-SX, Shimadzu). Each PSA was cut into rectangular specimens of 10 mm × 30 mm. Elongation rate: 500 mm/min. To observe the optical appearance of the deformed PSAs, the crossed polarizers were attached to the clamps.

### Cyclical Loading

The circular vibration test was conducted by an incubator shaker that shakes the substrate circularly (Excella E24, New Brunswick Scientific). One side of the specimens was attached to a nonvibrational wall with a spring to apply shear force, and the other end was attached to a stage that circularly vibrates using bolts and nuts to prevent torsions of specimens. The vibration test was conducted for up to 1 million cycles. The applied frequency of complex vibration was 5 Hz and the maximum applied force was 2 N. Slide displacements were measured from the length of the remaining adhesive layer on the substrate after the breakage. Measurements are conducted after stopping vibration during tests.

### Atomic Force Microscopy (AFM)

Surface morphology, adhesion property, and elastic modulus were attempted by using Dimension Icon AFM (Bruker) via peak force quantitative nanomechanical mapping (QNM) in accordance with the usual procedures.^25^ QNM was conducted with 512 × 512 samples/line at a scan rate of 1.0 Hz. The spring constant of the tip was 6.0 N/m and the tip radius was under 10 nm. Adhesion of the glass slide was measured from the force–distance curves.

### Shear Adhesion Behavior Under Human-Centric Extreme Environments

**Figure 1:**
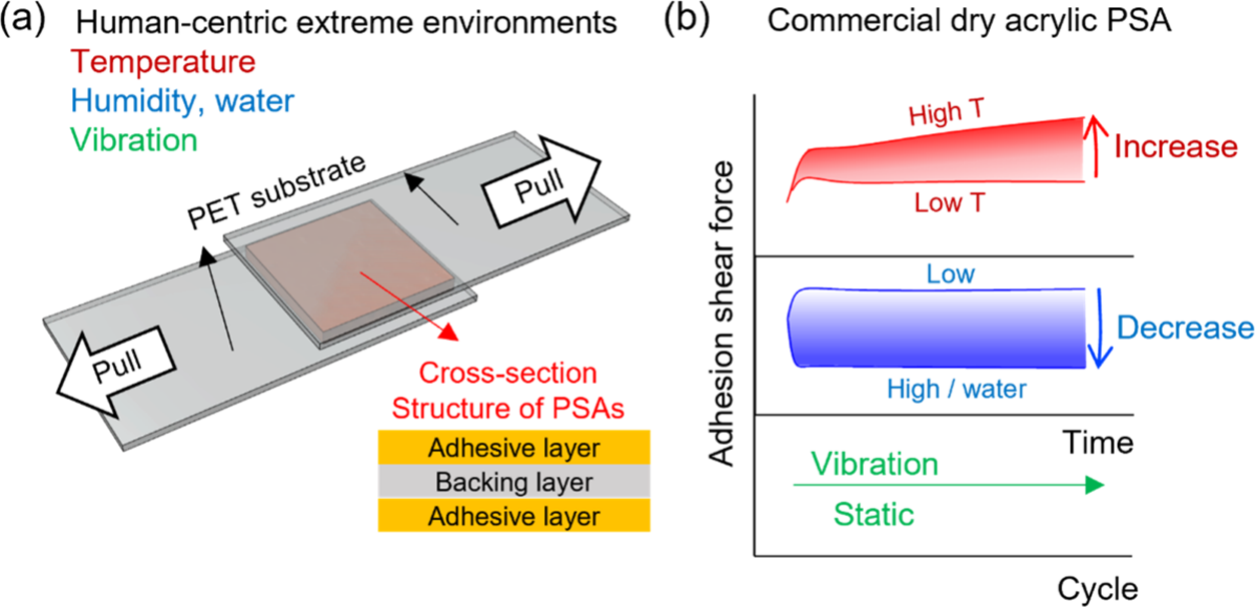
(a) Schematic illustration of the specimen arrangement for the peel test. (b) General trends of the adhesion strength variation (see the specific data discussed below) under different human-centric extreme environments as measured in this study.

After the specimens were clamped to a tensile tester, the load cell was used to measure the force for detaching the double-sided adhesive. Each PSA was cut into specimens of 0.5 in. by 0.5 in. and attached between two transparent poly(ethylene terephthalate) (PET) films to measure the force required to detach from the PET films (Figure 1a). After attaching to PET films, the specimens were gently pressed and left under ambient state for 2 h. The specimens were stored in convection oven, freezers, humidity chambers, and waters for different testing conditions. The specimens were then taken out and the shear adhesion strength was measured after 10 min of relaxing time. The shear adhesion strength was conducted by a universal tensile machine (EZ-SX, Shimadzu) with a 10 mm/min extension rate. The adhesive strength was calculated from the ultimate shear stress normalized to the dimensions of the adhesive contact area (Figure 1).

For the extreme environmental conditions, we simulated the environments in the real world including different temperatures (polar region, Death Valley, and body temperature), water conditions (normal water, artificial perspiration, synthetic seawater), different humidities (desert and tropics), and vibration/stress conditions (movable body parts) (Figure 1b). Specimens are stored in different environments, as listed in Table 1.

**Table 1:** Summary of Tested Environments

conditions	variations			
temperature (°C)	–70	–20	37	60
buffer (solution, pH)	4.5 (sweat)	6.8 (neutral)	8.1 (seawater)	
humidity (RH%)	5–10	40	90	
vibration	2 N, 5 Hz, up to 1,000,000 cycles			

For these studies, the specimens were stored in the convection oven (60 °C), hot plate with chamber (37 °C), and freezers (−70 and −20 °C). Next, the specimens were immersed under water simulating different liquid environments (pH 4.5, artificial perspiration; pH 6.8, deionized water; pH 8.1 synthetic seawater). To apply different humidity conditions, the specimens were stored in a sealed plastic chamber at a relative humidity (RH) of 5–10 RH%, 40 RH%, and 90 RH%. The periods of testing after storage at different conditions were 0 (initial), 1, 2, 5, 14, and 28 days.

## Results and Discussion

### Properties and Morphologies of PSAs

**Figure 2:**
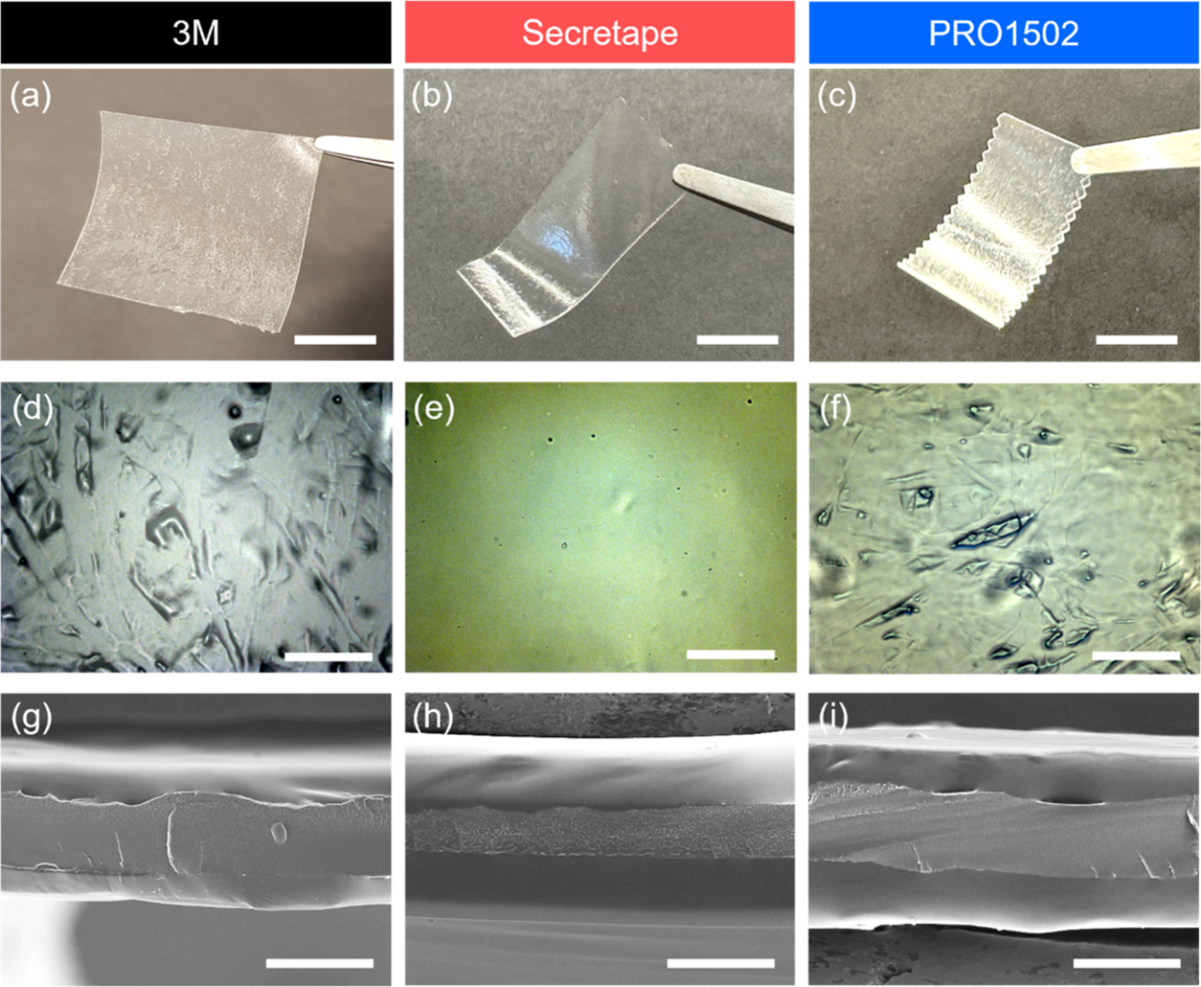
(a–c) Digital images, (d–f) optical microscopy, and (g–i) SEM micrographs of PSAs. Scale bars: (a–c) 10 mm, (d–f): 200 μm, and (g–i) 50 μm.

As is clear from the initial visual inspection, each PSA is opaque because of their rough and/or wrinkled surfaces (Figures 2a–f and S6a–d). The Secretape PSA has the highest optical transparency and a very smooth surface (Figure 2b,2e).

All of the PSAs are composed of three microscopic layers, densely packed: two external adhesive films act as the top and bottom layers separated by a central backing layer as can be seen from the cross-sectional SEM images (Figures 2g–i and S6e,f). The total thickness of the PSAs was within 160–180 μm and the 3 M tape with 130 μm total thickness was the thinnest among the adhesive tapes tested here.

As confirmed by FT-IR measurements, the chemical compositions of PSA layers are similar to all PSAs and include the combination of acrylate polymer matrix with some level of chemical modifications of aliphatic chains (Figure S7).^26−28^ Indeed, the main vibrational peaks of FT-IR spectra confirm the composition of acrylates: carbonyl (C=O), 1730 cm^–1^, C–O of ester group, 1230–1240 and 1160 cm^–1^, and methylene and methyl (CH_2_ and CH_3_), 2930 and 1455 cm^–1^. The peak positions are consistent with the analysis of the FT-IR spectra of the common commercial acrylic PSAs reported to date.^29^

### Chemical Stability of PSAs

**Figure 3:**
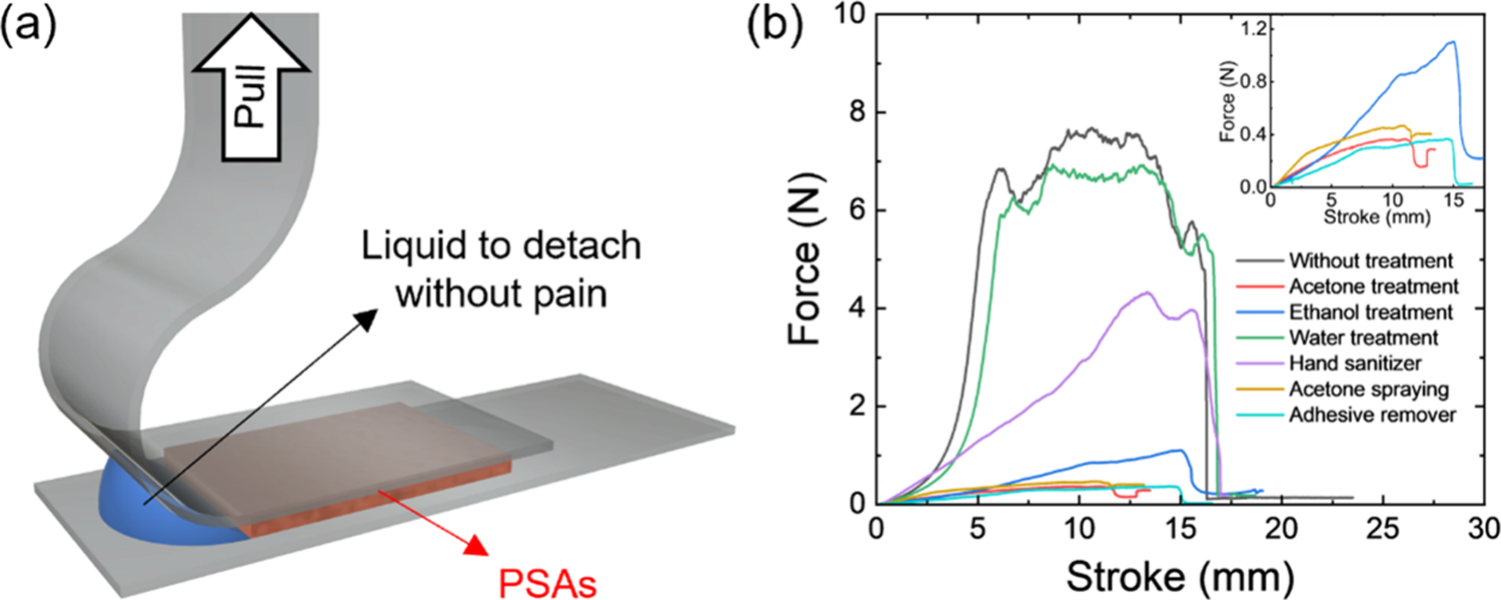
(a) Schematic illustration of the 90° adhesive peel test to verify the sustainability of the detaching force under different liquids. (b) Pulling forces after different treatments. The inset shows the low force regime.

The chemical stability of PSAs and the ability to release them were tested under various common solvents including water, acetone, ethanol, and merchandise (Figure 3a). One side of the specimen was attached to the substrate, and another side was clamped and vertically oriented to conduct a vertical peeling test (Figure 3a).

Next, as expected, the PSA performance was greatly affected by different solvent treatments (Figure 3b and Table S1). The adhesive-substrate boundary was treated with droplets of various solvents before the peeling test. A very minor decrease in adhesive peeling (5–20% of total strength) was observed for treatment with water and common commercial hand sanitizer liquids (isopropyl alcohol and water mix), thus indicating the stability of the adhesive contact under these conditions.

However, the adhesion strength dramatically reduces after treatment with common organic solvents, such as acetone or ethanol, and commercial liquid adhesive removers (Figure 3b and Table S1). Ethanol and acetone dramatically weaken the adhesive force and fully wet the PSAs in contrast to water (contact angle of 92°) (Figure S8). These adhesion behaviors and wettability mean that the interfacial tension between PSA and solvents plays a critical role in the sustained adhesion on plastic substrates.

Additionally, adhesion on the skin with and without oils from our body was studied (Figure S9). Finally, for preliminary testing, 3 M PSA of size 1 cm × 15 cm was gently attached to the clean arm after adding oil and kept for 5 min to stabilize the PSA. One side of the 3 M PSA was clamped and pulled in a perpendicular direction. The adhesion of 3 M PSA on oily skin decreased by half in comparison to tape adhesion to clean skin.

Overall, these results confirm that easy debonding can be readily initiated by organic solvent treatments, but adhesive contact remains very stable under common daily treatments such as pure water washing and disinfecting.

### Mechanical Properties of PSAs

**Figure 4:**
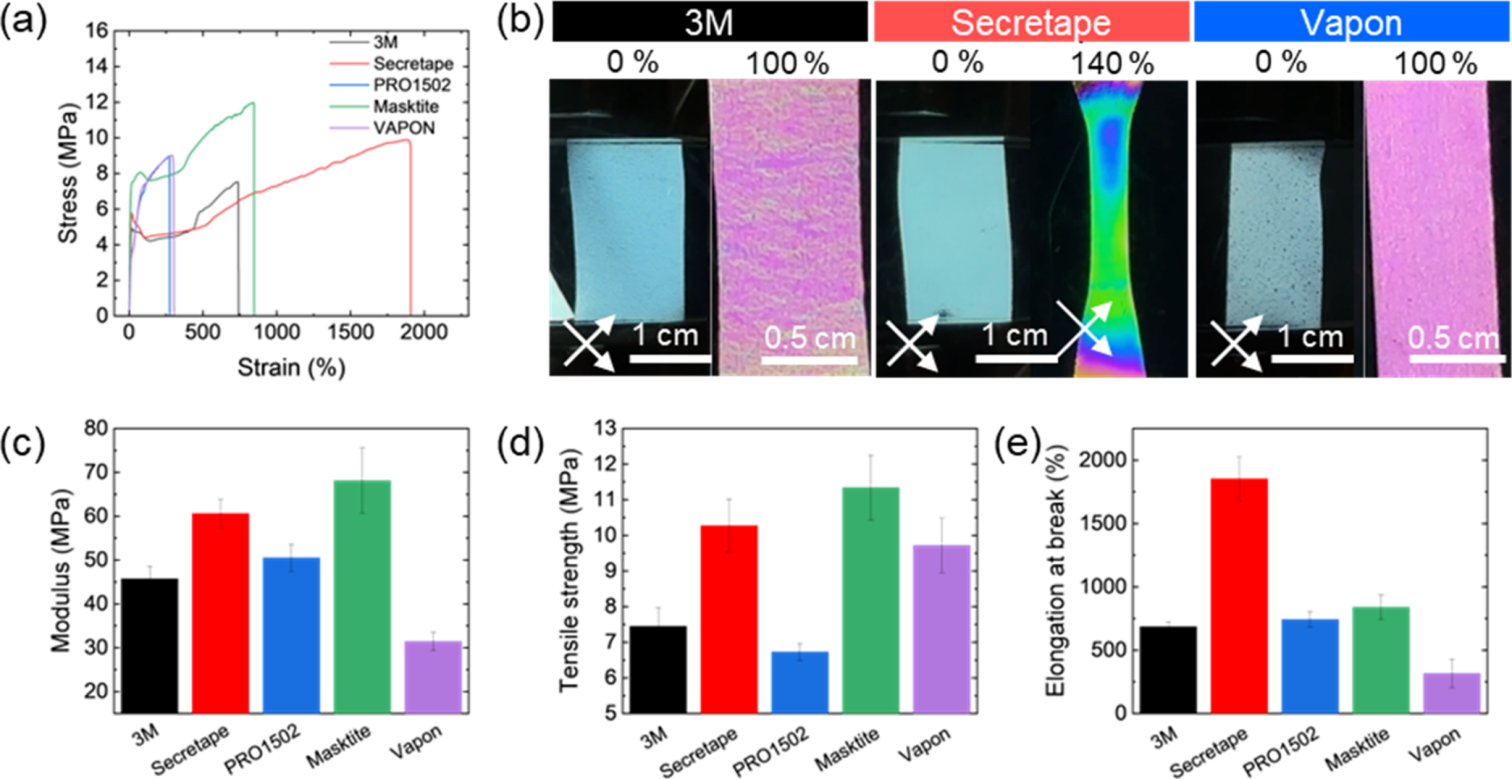
Mechanical performance of PSA films: (a) stress–strain data; (b) cross-polarized images of PSAs before and after elongation. Summary of mechanical performance: (c) Young’s modulus, (d) tensile strength, and (e) elongation at break.

The stress–strain measurements for all PSA films show a common deformational behavior that is typical for adhesive elastomeric polymeric materials (Figure 4). The stress gradually increases to 5–11 MPa at high ultimate strain (Figures 4a, S10 and S11). The highest tensile strength reached 11 MPa for Masktite tape (Figure 4c,d).^29,30^ All PSA films are very stretchable, with the highest elongation at break reaching extremely high values before the final break at 1800% elongation for the Secretape PSA (Figure 4e).

The elastic modulus varies from 30 to 70 MPa for different PSA films, with the highest value reaching 65–70 MPa for Secretape and Masktite PSAs. The elastic modulus of PSA is lower than that of the common low-density polyethylene (LDPE), 100–300 MPa,^31^ which is composed of a central carrier layer due to the presence of much softer acrylic layers with a much lower modulus.^32^

Some peculiar drop in stress was observed at intermediate strains for several PSAs due to the delamination and breakage of different layers (Figure 4a). The central carrier layer that has a higher mechanical stiffness to maintain the shape of the PSA contributes to the former part of the stress–strain curves, and the latter parts are contributed by the adhesive layers. Even if the central LDPE carrier layer breaks first, the external elastomeric adhesive layers can maintain the overall tape integrity without full failure. Therefore, ultimately, catastrophic failure occurs at much higher stretching (Figure 4a).

Next, we tested how strain rates affect the mechanical properties, with common strain rates selected as 50, 250, and 500 mm/min (Figure S12). Overall, we observed modest strain rate dependencies of within 10–15%: as the strain rate increases, tensile strength increases while elongation at break decreases. In addition, the first peaks of stress–strain curves increase as the strain rate increases, which indicates that the materials become somewhat stiffer when the strain rate increases.

To visualize the elongation behavior of PSA films, the tensile testing was further conducted with optical observation under crossed polarizers for selected adhesives (Figure 4b). We depicted 100% strain and 140% strain for clear visualization of optical variations. At modest elongation, we observed a common necking process and uniform coloration of the Secretape PSA due to uniform and unidirectional orientation of PSA polymer layers with uniform elongation of polymer chains (Figure 4b). On the other hand, intense wrinkle formation was observed for other PSAs due to delamination of adhesive layers from the central carrier layer and nonaffine deformation of different layers (Figures 2 and 4b).

**Figure 5:**
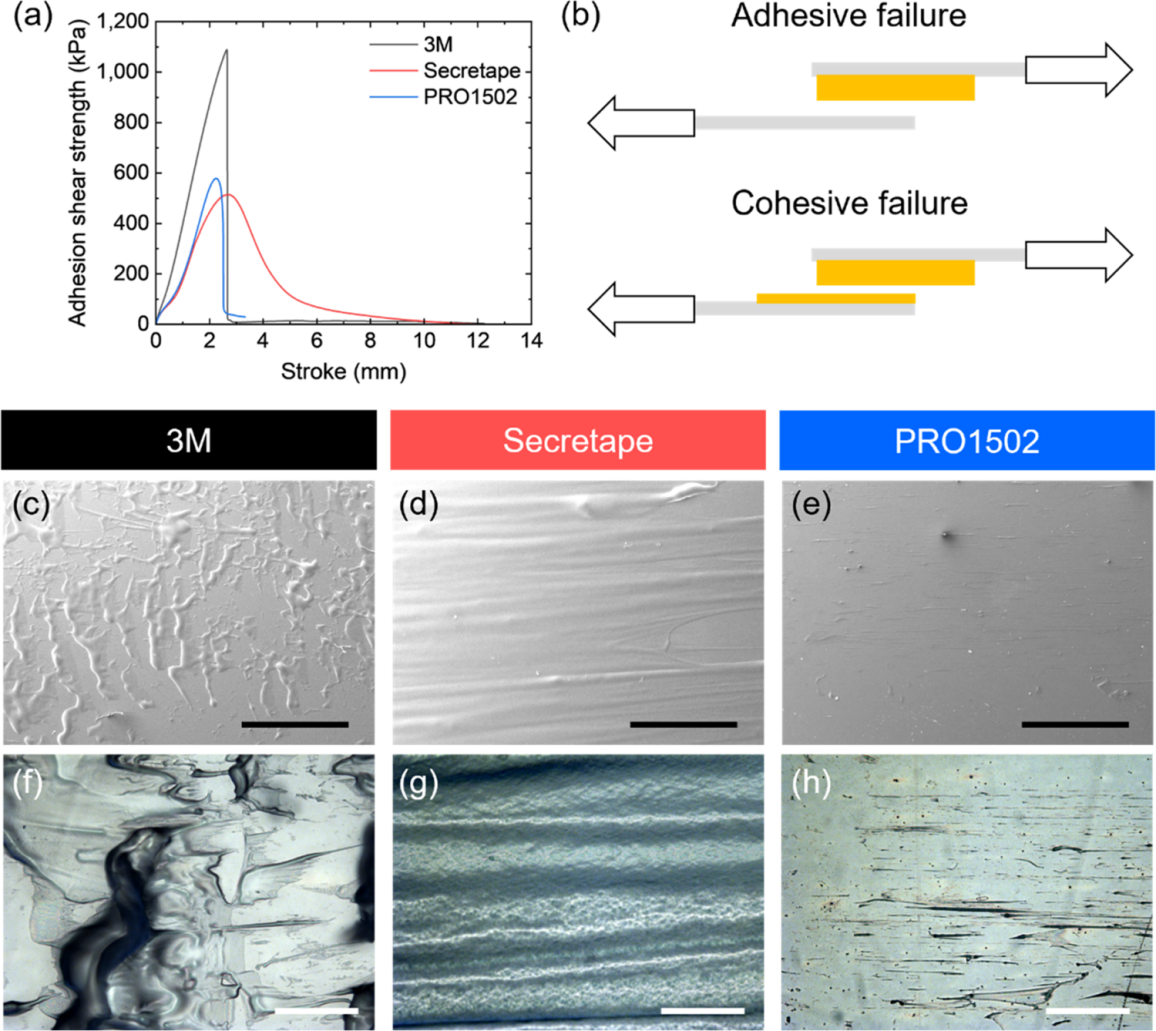
(a) Adhesion shear strength-stroke curves of PSAs. (b) Schematics of shear-induced failures. Materials’ residuals after detachment: SEM (c–e) and optical micrographs (f–h) of the substrates after failure. Scale bars: (c–e) 2 mm; (f–h) 200 μm.

Generally, the 3 M PSA possesses the highest Young’s modulus, and the Secretape tape shows the highest elongation at break. We conducted a direct comparison of these tapes to the intermediately performing tape, PRO1502. For these selected PSAs, lap shear tests were conducted to measure the adhesion shear strength of adhesive tapes after different treatments as discussed below (Figures 5a and S13). As observed, different adhesive strengths between the substrate and PSA induce sharp or continuous detachment from the substrates (Figure 5b). As known, adhesive failure occurs when the bond between the adhesive layer and substrate is stronger than the bond between the adhesive layer and substrate due to the weak adhesive strength and/or interaction between them. In contrast, cohesive failure is facilitated by the strong interaction between the adhesive layer and substrate and/or weak mechanical resistance of the adhesive layer.

For the 3 M PSA with high adhesive strengths and elastic modulus, residual adhesive material remains on the substrate that delaminated from the central carrier layer, thus indicating cohesive failure (Figure 5c,5f). The high adhesion shear strength of 3 M PSAs implies that 3 M PSAs have a strong bonding to the substrate that induces a sharp drop and breakage of the adhesive layer. The Secretape PSA with lower adhesion shear strength possesses cohesive failure (Figure 5d,5g). At high stretching levels, the adhesive layer is mostly elongated along the pull direction for Secretape specimens, which induces continuous detachment from the substrate.

On the other hand, clean adhesive failure was observed for PRO1502 PSAs. The low adhesive shear strength and small elongation of PRO1502 PSAs induce a sharp drop of adhesion and detachment. Indeed, clear PET substrate surfaces were observed after complete adhesive tape detachment (Figure 5e,5h).

Additionally, different shear rates were applied to test the rate dependence of adhesive tapes (Figure S14). Indeed, the PSAs become slightly stiffer at higher shearing rates although the increase is very modest and confirms the consistent mechanisms of adhesive failures for common shear rates for general mechanical testing.^33^

AFM-based quantitative nanomechanical mapping (QNM) was attempted for nanoscale pull testing of 3 M PSA surfaces (Figure S15). The QNM images show surface morphology, apparent modulus, and adhesion distribution (Figure S15d–f). AFM mapping shows a relatively uniform local surface morphology with nanoscale (below 100 nm) inclusions and bumps with elevated stiffness and decreased adhesion.

However, attempts to measure and quantify local adhesion consistently were not successful due to the extremely high pull-off forces and high indentation of very soft and highly attractive acrylic materials. Indeed, even the stiff AFM cantilevers were readily broken (Figure S15a). The full-off forces of 3 M PSA were estimated to be 3800-fold higher than those of the control glass surface as estimated from the occasional successful force–distance data (Figure S15b,c).

## Adhesive Behavior at Variable Extreme External Conditions

### PSA Performance After Treatments at Various Temperatures

**Figure 6:**
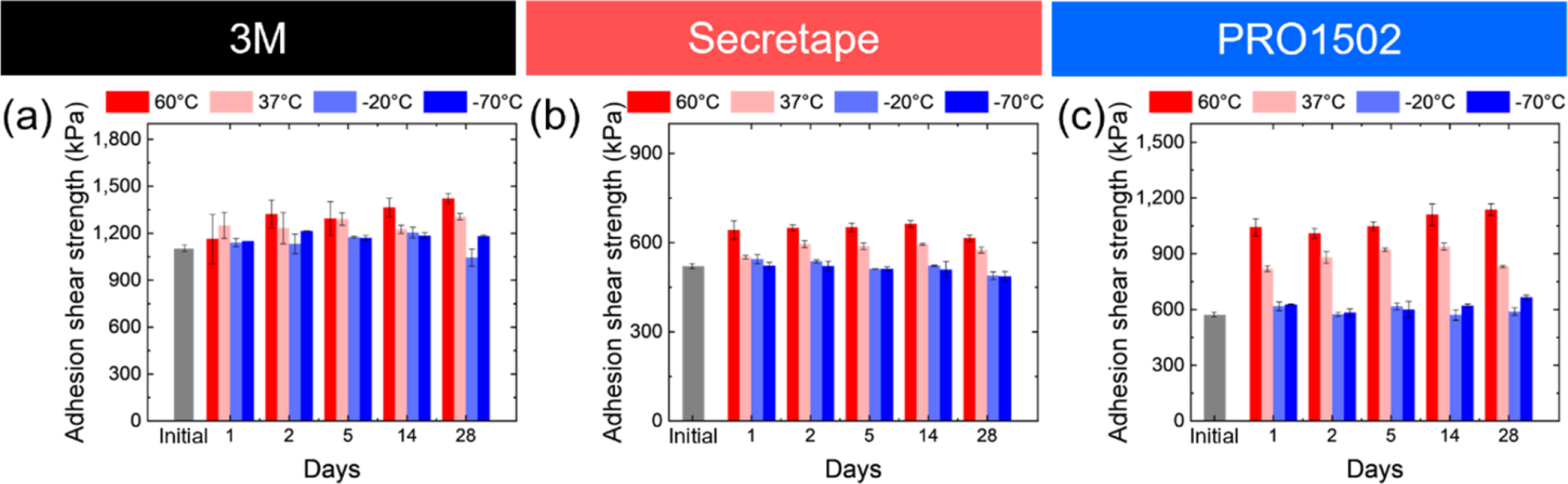
(a–c) Adhesive strength of PSAs under different thermal treatments: (a) 3M, (b) Secretape, and (c) PRO1502.

**Figure 7:**
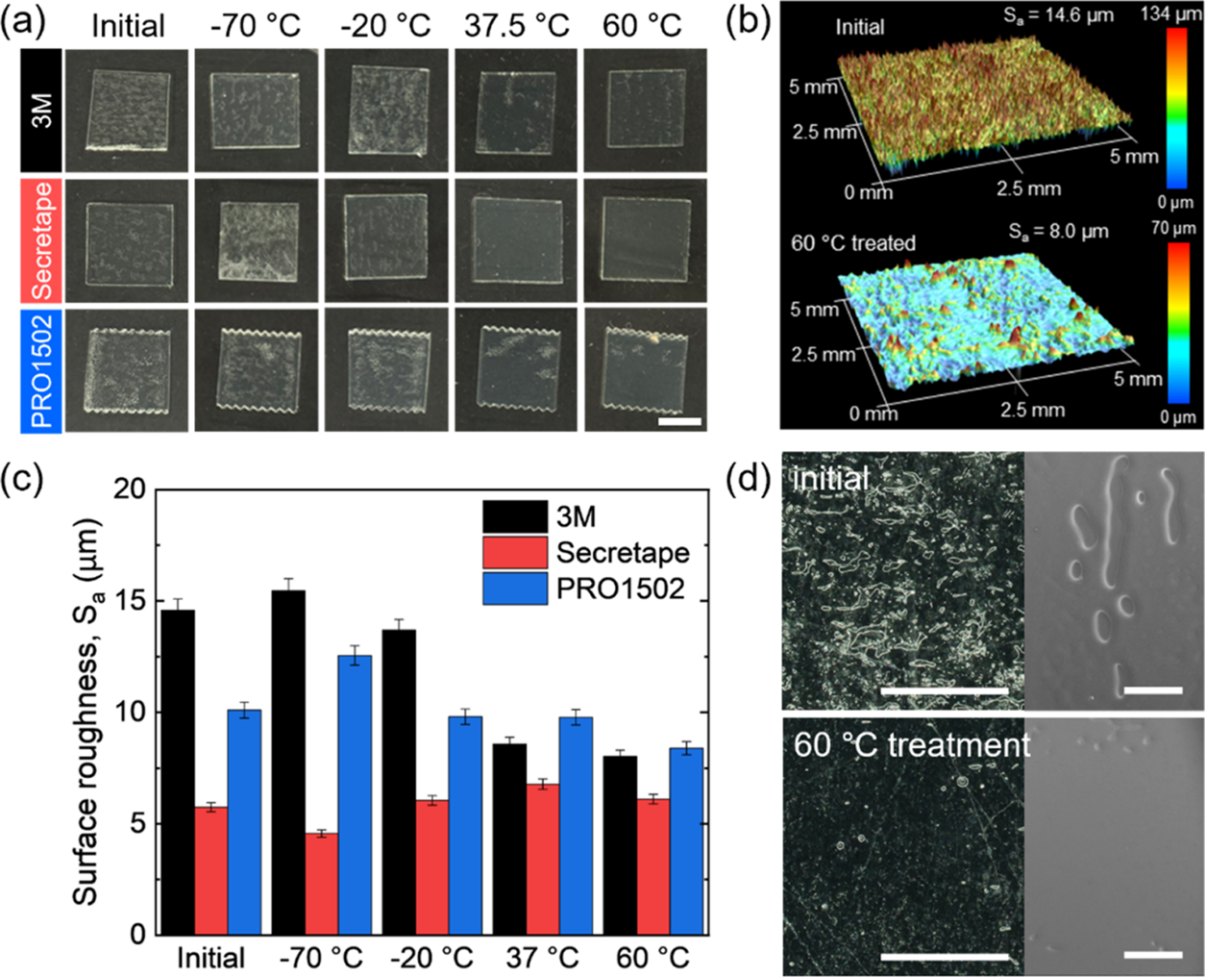
(a) Photographs of PSAs treated under different temperature conditions. Scale bar: 1 cm. (b) 3D surface profiles before/after 60 °C treatment of 3 M PSA. (c) Surface roughness (*S*_a_) of PSAs treated under different temperature conditions. (d) Photographs and SEM micrographs of 3 M PSA before/after 60 °C treatment. Scale bars: 500 μm.

For the three selected PSAs discussed above, we followed long-term adhesive strength monitoring at different temperature treatments: close to “normal” skin conditions (37 °C), elevated temperature related to possible extreme summer temperatures in deserts (60 °C), and low as well as very low temperatures relevant to common winter conditions and extreme polar conditions (−20 and −70 °C) (Figures 6 and 7).

As was observed, the adhesion strength of all PSAs studied here increases significantly (from 20 to 70%) after storage at elevated temperatures, with a general trend toward higher adhesive strength occurring at longer thermal treatment times (Figure 6).

In contrast, thermal treatment at different low temperatures, −20 and −70 °C, does not affect significantly the adhesion strength of the adhesive tapes at any prolonged storage time (within 5–10%) (Figure 6). The stability of chemical composition under these temperature conditions is confirmed by the absence of any significant changes in intensities or shifts in all characteristic FT-IR peaks for all PSAs after thermal treatment (Figure S16a–c).

Overall, we suggest that the increase in adhesion strength after storage with elevated temperatures can be mostly related to the gradual increase in the contact area of softened materials without significant changes in the chemical composition caused by additional cross-linking or crystallization.

Moreover, thermal post-treatment at elevated temperatures resulted in transparency because of reduced light scattering and optical losses (Figures 7a,7b, S17 and Table S2). Furthermore, the PSAs become much smoother, thus, also reducing light scattering. Indeed, the surface roughness decreased drastically, by 20–40% after annealing at elevated temperatures. E.g., the surface roughness of the 3 M PSA decreased most dramatically from 14.6 to 8.0 μm when stored at 60 °C and the surface roughness of PRO1502 PSA also decreased from 10.1 to 8.4 μm (Figure 7c). In contrast, only small changes were observed for Secretape PSAs with intermediate adhesive performance.

Additionally, as indicated by optical microscopy and SEM of free surfaces of PSA films and interfaces with substrates after thermal treatment, the surface inhomogeneities, interfacial pores, and cavities between the substrate and PSA film diminished drastically (Figures 7d and S18). We suggest that this overall smoothening of the films is facilitated by the significant softening of thin films at elevated temperature that causes the spreading of the mechanical contact area and possibly degassing of some cavities. In contrast, at low-temperature treatment, high mechanical stiffness restricts changes in the conformal contact area with the substrate and prevents interfacial pore degassing.

### PSA Performance after Various Aqueous Environments

**Figure 8:**
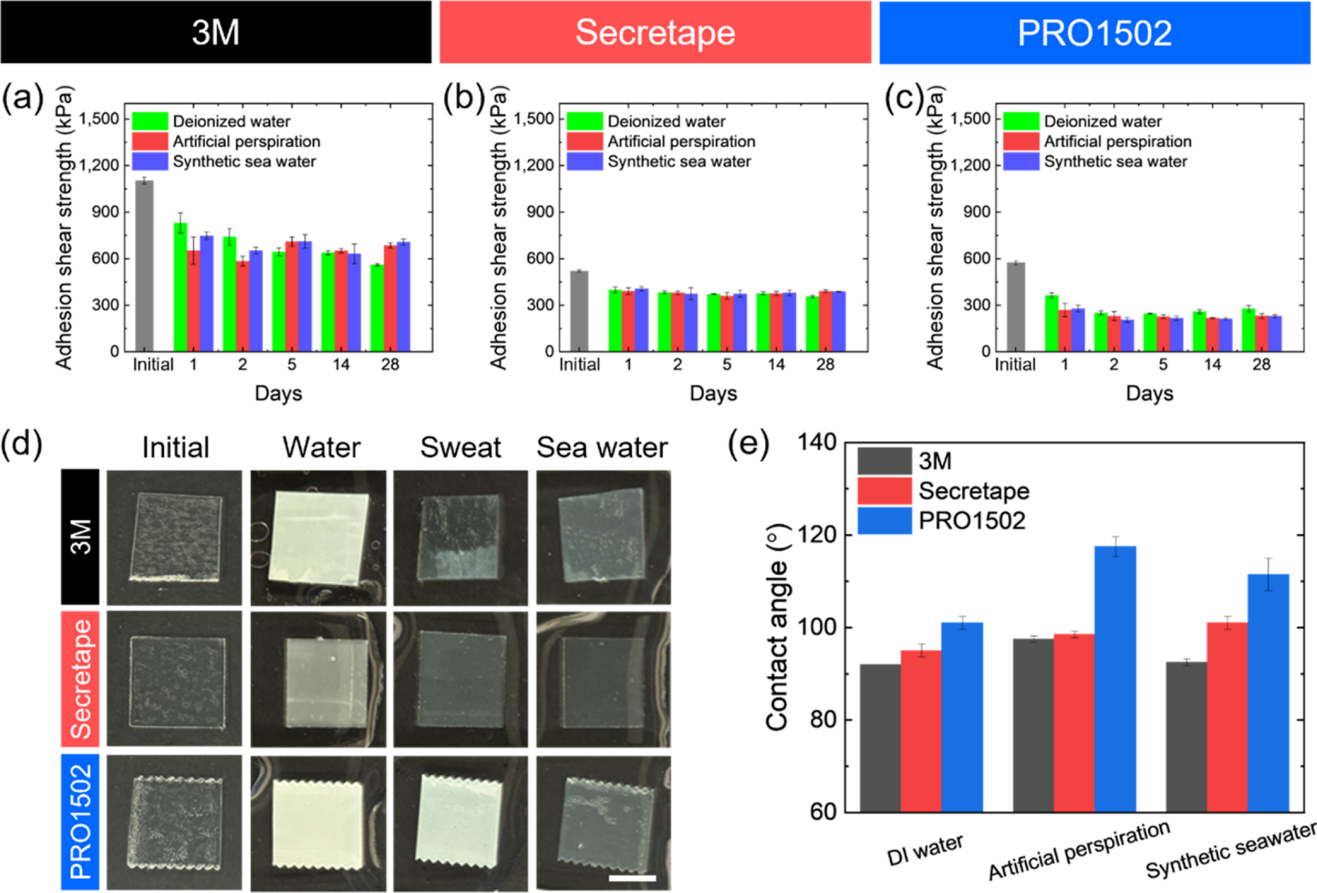
(a–c) Adhesion shear strength of PSAs under different water treatments. (a) 3M, (b) Secretape, and (c) PRO1502. (d) Photographs of three different PSAs under different liquid treatment conditions. Scale bar for all images: 1 cm. (e) Contact angles of dried PSAs treated with different liquids.

Next, to elucidate the adhesive strength changes in aqueous conditions relevant to daily human existence, the PSAs were immersed in neutral water (shown as showering), artificial sweat (shown as exercising), and synthetic seawater (shown as swimming) (Figure 8).

The results of the lap shear test of PSAs treated with water show a gradual, continuous sliding, while the initial PSA showed a sharp drop of adhesion (Figure S19). As we observed, for all PSAs the adhesion shear strength decreased gradually within the initial 2 days of aqueous treatment and then remained mostly unchanged for a longer treatment time, up to 28 days even if the PSAs became swollen (Figure 8a–c). Indeed, the increased hydroxyl bond (−OH) peak, around 3300 cm^–1^, and H–O–H scissoring peak, at 1640 cm^–1^, in the FT-IR spectra confirm that the PSA films are highly swollen (Figure S16d–f).^34^ On the other hand, all PSAs become hazy in comparison with the original specimens because of the nonuniform swelling of the adhesive layers that induces excessive light scattering (Figure 8d).

Finally, we measured the wettability of PSA films by measuring the contact angle (Figures 8e and S20). The contact angle within 90 to 100° is common for hydrophobic materials and is expected for the modified acrylic-based PSA materials studied here. After treatment with artificial perspiration and synthetic seawater, the contact angle somewhat increases to 100 – 120° (Figure 8e). This increase can be related to additional surface roughening and crumpling after a quick removal of excess liquid. Therefore, we conclude that the adhesive strength reduction after liquid treatments is caused by the initial excessive swelling and surface crumpling after quick drying of adhesive layers that obstruct the interface between the adhesive layer and substrate.

### PSA Performance at Various Humidity Conditions

**Figure 9:**
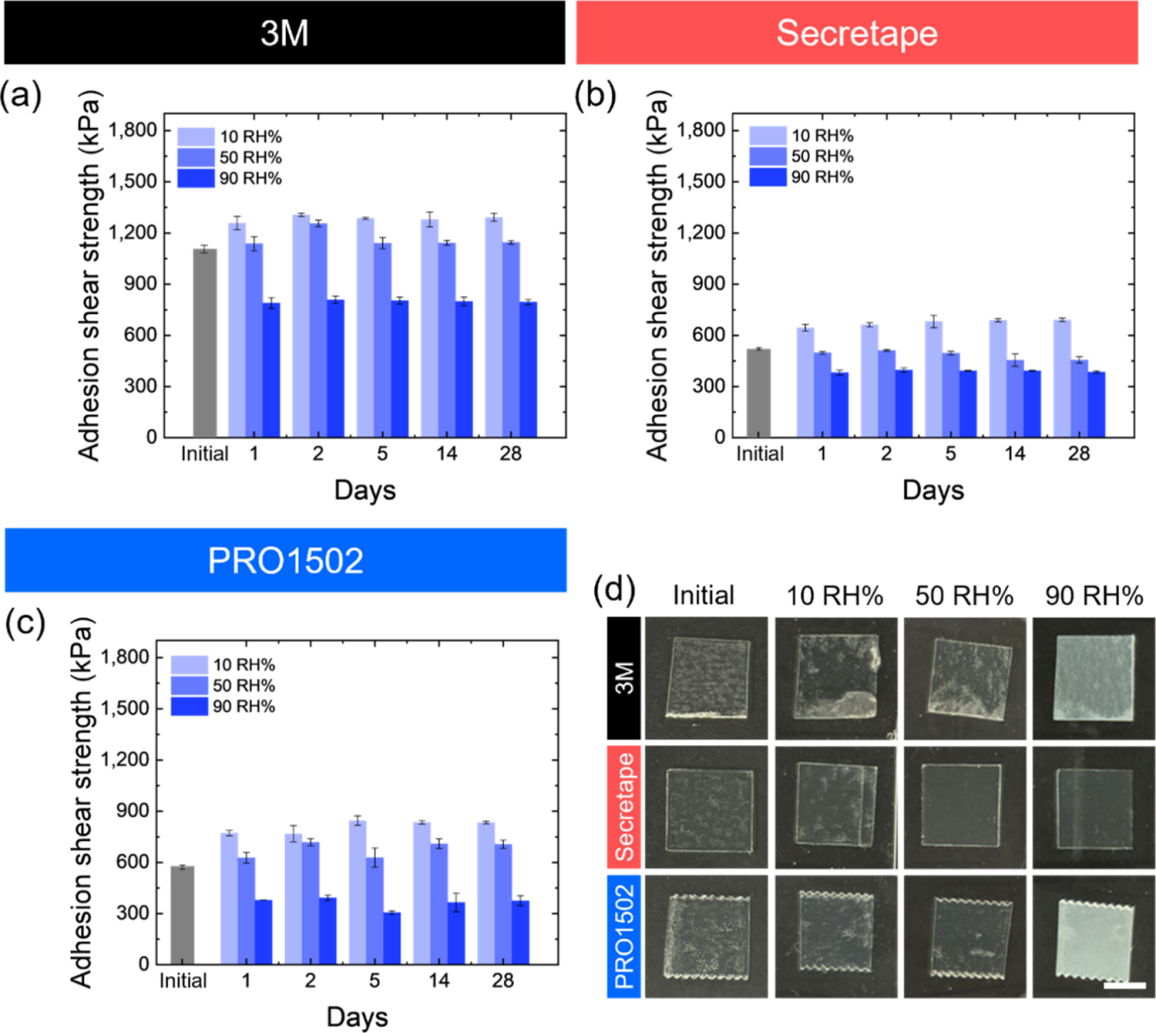
(a–c) Adhesive strength of PSAs under different humidity treatments: (a) 3M, (b) Secretape, and (c) PRO1502 tapes. (d) Photographs of three different PSAs after treatment with different humidity conditions. Scale bar: 1 cm.

Storage under different humidity conditions affects the adhesive strength similarly to that of the liquid treatments discussed above (Figure 9). After storage at high humidity, the adhesion strength decreases significantly, by 40–60%. However, storage at a reduced humidity of 10% and ambient humidity does not affect the adhesion strength significantly, with a slight increase observed for some cases (Figure 9). Finally, storage at the highest humidity causes the PSA films to become opaque, while storage under ambient and low-humidity conditions does not affect the film opacity significantly (Figure 9d).

### Optical Properties of PSAs

**Figure 10:**
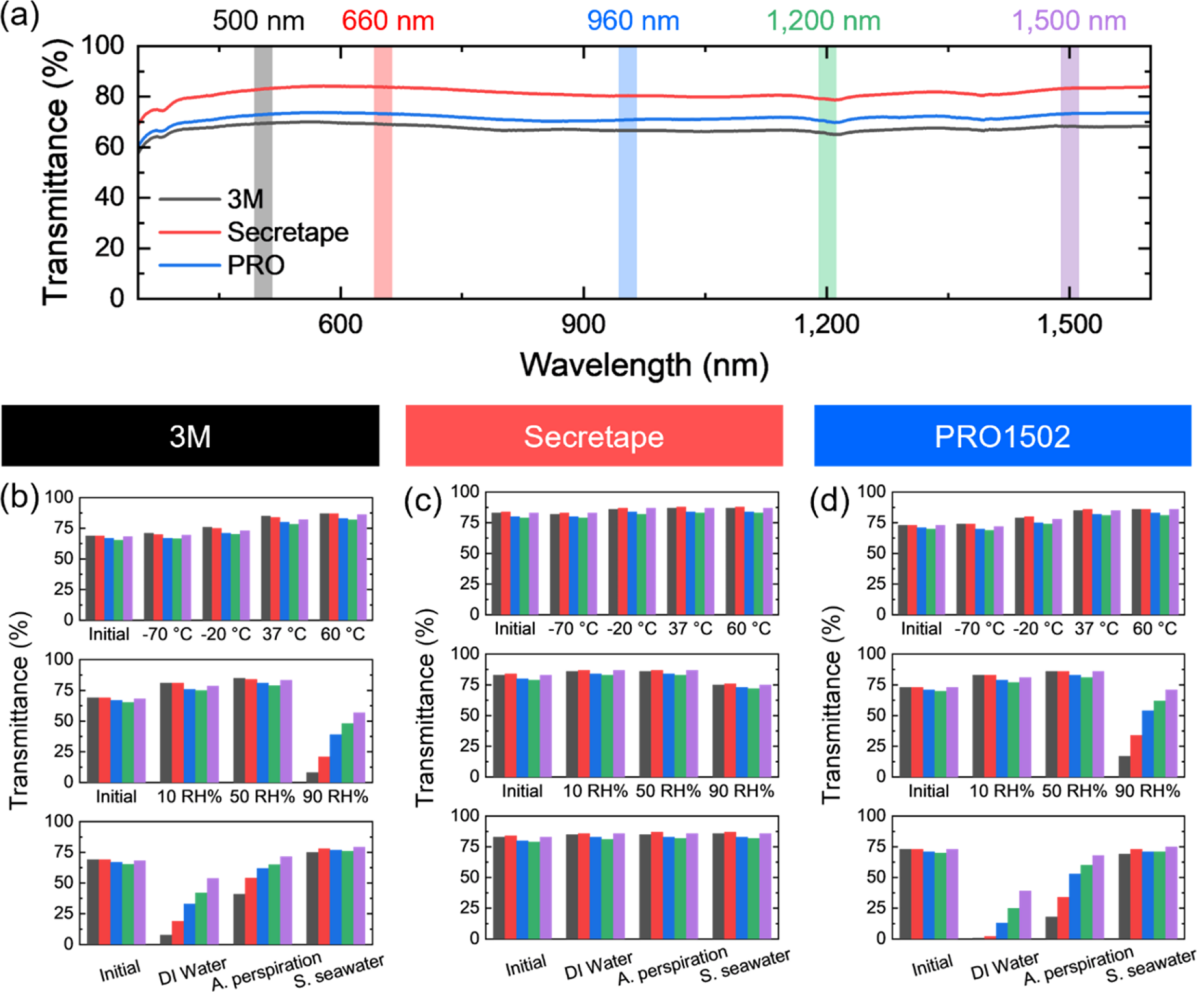
(a) UV–vis-NIR spectroscopy and (b–d) transmittance of PSAs under different environmental treatments. (b–d) top: temperatures, middle: humidities, and bottom: different liquids.

As known, human health monitoring devices such as electrooculography and photoplethysmogram use optical channels for motion detection with different light-emitting diode (LED) lasers.^35−37^ The most popular choices of wavelengths for these devices are green light at 500–550 nm, red light at 650–700 nm, near-infrared light at 900 nm, or even infrared light within 1200–1500 nm. Therefore, the variation of the optical transmittance of PSAs under variable environmental conditions was monitored at the relevant characteristic wavelengths of 500, 660, 960, 1200, and 1500 nm in order to evaluate their sustainability (Figures 10 and S21).

First, it is worth noting that all original PSAs show high optical transparency, ranging from 65 to 85%, in the whole spectral range and across all wavelengths tested here (Figure 10a). This transparency is sufficient for optical reading sensors. Among all tapes, Secretape has the highest transparency because of its lower light-scattering losses due to a smooth surface and low pore concentration as discussed above. Second, under different environments, the transmittance remains very high for this tape (Figure 10c).

The other tapes hold their high transmittance at lower temperatures and become more transparent under thermal treatment at elevated temperatures due to PSA swelling, pores and cavities healing, and overall smoothening of the PSA surfaces (Figure 10). Finally, the tapes became more opaque when immersed in different liquids due to increased surface crumpling after drying (Figure 10b,10d). Overall, all tapes studied here demonstrate sustainable high optical transmittance that is acceptable for optical sensor monitoring under real-life conditions.

### Cyclic Mechanical Loading of PSAs

**Figure 11:**
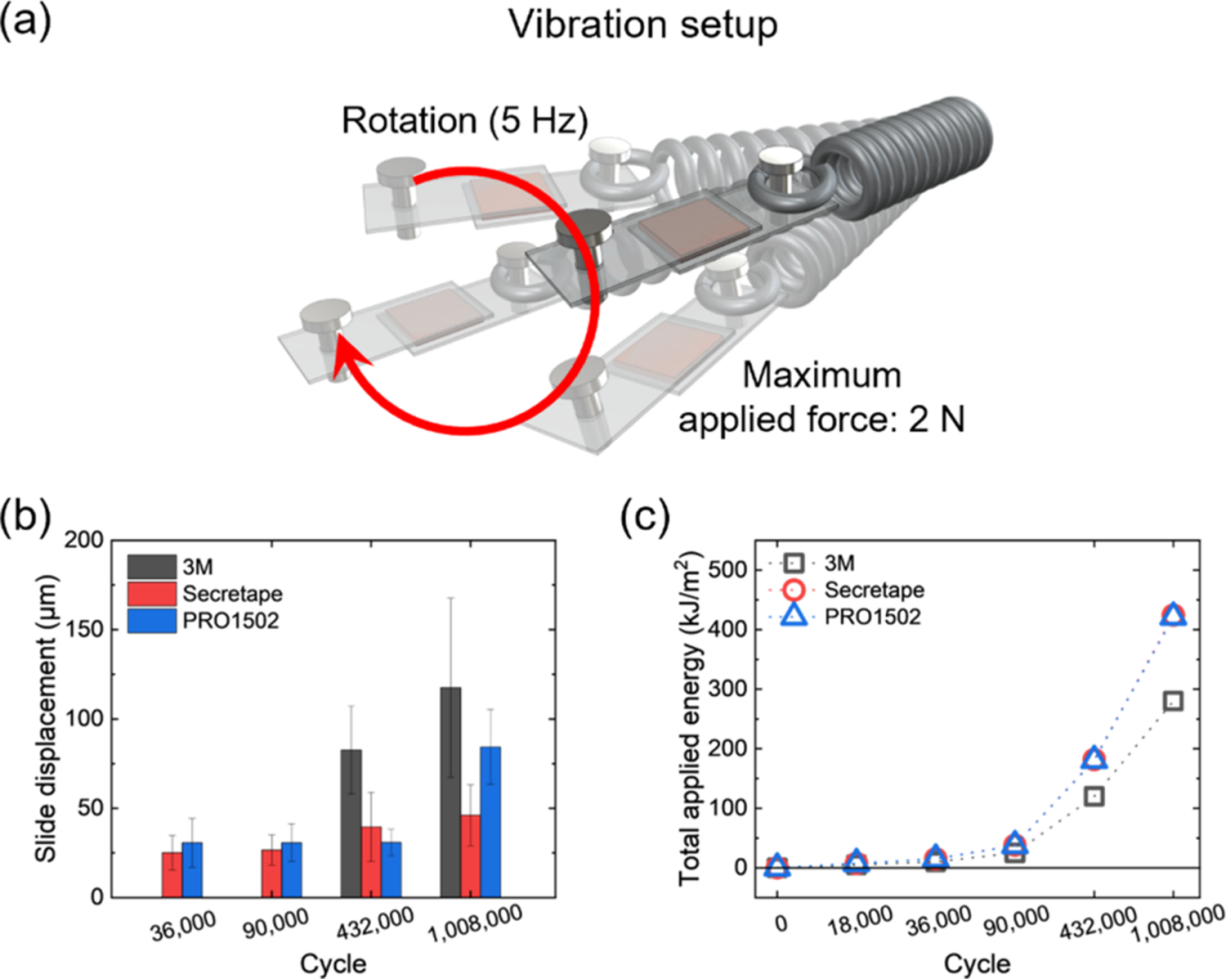
(a) Schematic illustration of the vibration setup for cyclical testing. (b) Slide displacements of each PSA during the vibration test. Slide displacement of PSAs according to the vibration cycle. (c) Total applied energy to PSAs for different numbers of vibration cycles.

Finally, we tested the sustainability of adhesive performance under the cyclical mechanical load common for using these tapes for wearable devices (Figure 11). In our life, the things on our body move due to internal and external stimuli, including our body motion and changes in the vibrational surroundings. The wearable devices on the body must be stably attached to maintain their capabilities. To simulate these motions for stability evaluation of PSAs on the substrate, circular cyclic mechanical loading was applied, which is a combination of multidirectional loading. A frequency of 5 Hz was applied by adapting the average step frequency of sprinters and the common building vibrational pattern.^38^ Circular movement is a combination of all directions that represent complex body motion.

First, we tested the ultimate failure of all tapes under cyclical circular loads within 1.5 to 2.5 N loads and observed long-term stability up to a million loading cycles (Figure S22). One side of the spring was attached to the wall by glue, but it easily detached from the wall before reaching half a million cycles. Thus, these holders were replaced by more robust printed spring holders. In this testing, all of the displacement of adhesive joints was measured with a 200 μm vibration amplitude (Figure 11b).

All PSAs showed high mechanical sustainability extended to one million cycles without failure, which exceeds the expected monitoring lifetime (several days) of wearable technologies. The lowest ultimate displacement of 50 μm after a million loading cycles was observed for Secretape and somewhat higher, up to 120 μm, for the other tapes tested here. Furthermore, the total mechanical energy to achieve this displacement was similar for all tapes and was estimated to be within 250–400 kJ/m^2^ (Figure 11c).

## Conclusions

In this study, different common double-sided pressure-sensitive adhesive materials were analyzed in terms of their morphology, adhesive strength, optical properties, and mechanical performance for their sustainable applications for long-term mounting wearable health-monitoring sensors and devices. As was demonstrated, trilayered tapes with microscopic thickness within 100–200 μm are composed of a strong central layer and outer acrylic adhesive layers and possess modest surface roughness, porous morphology, and high optical transmittance. The vast majority of adhesive tapes studied here are highly stretchable with ultimate strains within 500–2000% and show a traditional elastomeric behavior with elastic moduli within 40–70 MPa, comparable to those of common elastomers and human skin.

Furthermore, PSA films with high-adhesive performance were selected for continuous testing under challenging human-centric environmental conditions including various low/high temperatures, humidity levels, various aqueous liquid exposures, and complex cyclical loading, for periods up to 4 weeks. The thermal treatment above human body temperature induces expansion of conformal mechanical contact with the substrate, increasing both the adhesion strength and optical transmittance in all adhesive materials due to pore healing and surface smoothening. These tapes maintain high adhesion strength and optical transmittance at low-temperature treatment as well. When PSA films are immersed in different liquids or exposed to a high-humidity environment, the adhesion strength decreases because of excessive swelling and reduced interactions between the substrate and swollen adhesive layers. In addition, drying of swollen PSAs results in higher surface roughness because of crumpling during quick deswelling that reduces the optical transparency and affects the adhesive performance of liquid-treated PSA materials. The PSA films after thermal annealing at elevated temperatures show increased adhesion shear strength and remain mostly unchanged during freezing treatment.

In terms of sustainable long-term cyclical mechanical performance relevant to the mounting of wearable devices, all PSAs exhibited a minor shape distortion during one million cycles, indicating an extremely high stability under cyclical vibrations that is the equivalent of several days of services. The PSAs with dominating cohesive failure and gradual trilayer delamination show the highest adhesion shear strength and sustainability in all human-centric extreme environments.

Overall, we demonstrated that the adhesive performance of pressure-sensitive triple-layered polymeric materials under various challenging human-centric environmental conditions can be directly related to the observed changes in their overall morphologies, surface homogeneity, surface roughness, layer delamination, swelling state, and variation of the mechanical contact area. The trends in structure-properties relationships suggested here for a variety of popular PSA adhesive materials constitute important contributions to the development of consistent rules of materials design for wearable human sensors for important human health and performance monitoring in their applications under challenging environmental conditions.

## Supporting Information Available

Information on each PSA; digital images; OM and SEM micrographs of PSA surfaces; FT-IR before/after treatment of each PSA; contact angle of PSAs; pull-test on skin with/without oil; stress–strain curves of PSAs with different rates; setup for shear test and adhesion shear strength curves before/after treatment; AFM QNM for surface morphologies; modulus and adhesion; 3D surface profiles of PSAs before/after thermal treatment; UV–vis spectroscopy of PSAs before/after treatments; analysis of circular vibration (PDF)
