# Supplementary material for: Double-Sided Pressure-Sensitive Adhesive Materials under Human-Centric Extreme Environments
Source: ACS Appl Mater Interfaces. 2024 Sep 2;16(36):48257–68. doi: 10.1021/acsami.4c09327 (PMC11403543; doi:10.1021/acsami.4c09327)
Supplement: Supplementary file 1 — am4c09327_si_001.pdf [file am4c09327_si_001.pdf]

Supporting Information

Double-sided Pressure-Sensitive Adhesive Materials under Human-Centric Extreme Environments

Jisoo Jeon[1+], Jinyoung Kim[1+], Sehyun Park[1], Gwendolyn Bryan[2,3], Timothy J. Broderick[2], Morley Stone[2], Vladimir V. Tsukruk[1,2]*

[1] School of Materials Science and Engineering, Georgia Institute of Technology, Atlanta, GA 30332, USA

[2] Florida Institute for Human and Machine Cognition, Pensacola, FL 32502, USA

[3] Department of Intelligent Systems and Robotics, University of West Florida, Pensacola, FL 32514, USA

[+]These authors have equal contributions to this paper.

*Corresponding author e-mail: vladimir@mse.gatech.edu

Materials data

3M™ Double Coated Medical Polyethylene Film Tape
Effective: December 2020   Supersedes all previous versions

Features and Benefits:

- Non-Sensitizing per ISO:10993-10
- Easy to Apply[1]
- Translucent, Flexible LDPE Film Tape
- EtO & Irradiation Sterilization Compatible[1]
- Medium Weight Release Liner
- Good for Medium-Term Wear on Skin[1]

Composition: Tape is typically ~4.9 mils [~0.12 mm] thick without release liner.

**A**-Paper Liner	60 lbs. Bleached Kraft Paper, DC Si Rel. (3.5 mils/0.09 mm)
**B**-Acr Adhesive	Tackified Acrylic-based Pressure Sensitive Adhesive
**C**-LDPE Carrier	3 mil (0.08 mm) Translucent Polyethylene Film Carrier
**D**-Acr Adhesive	Tackified Acrylic-based PSA (Faceside)

Figure S1. Specifications of 3M PSA tape.

Uplift Secret Fashion Clothing Tape Double Sided - Clear Tape for Clothes, Body, and All Skin Shades (100 Strips)

Figure S2. Data on Secretape.

Pro 1502 Self-adhering Face Mask Tape 1/2in (12mm) x 20ft Clear Double Coated Medical, Direct-to-Skin, Fabric to skin. skin friendly adhesive, fluid resistant, Easy to apply, Can reposition/reapply

Figure S3. PRO1502 tape characteristics.

MaskTite – Face Mask Tape, No Fogging Glasses. No Slipping Masks. No Gaps. Made in USA. Gentle, Medical-Grade, Hypoallergenic, Latex Free, Double-Sided Tape 36 Precut Strips. Works with Any Mask.

Figure S4. Masktite tape data.

Vapon Double Sided Medical Grade Adhesive Tape Roll, 1 Inches x 108 Inches-Clear

Figure S5. Vapon tape characteristics.

Experimental results

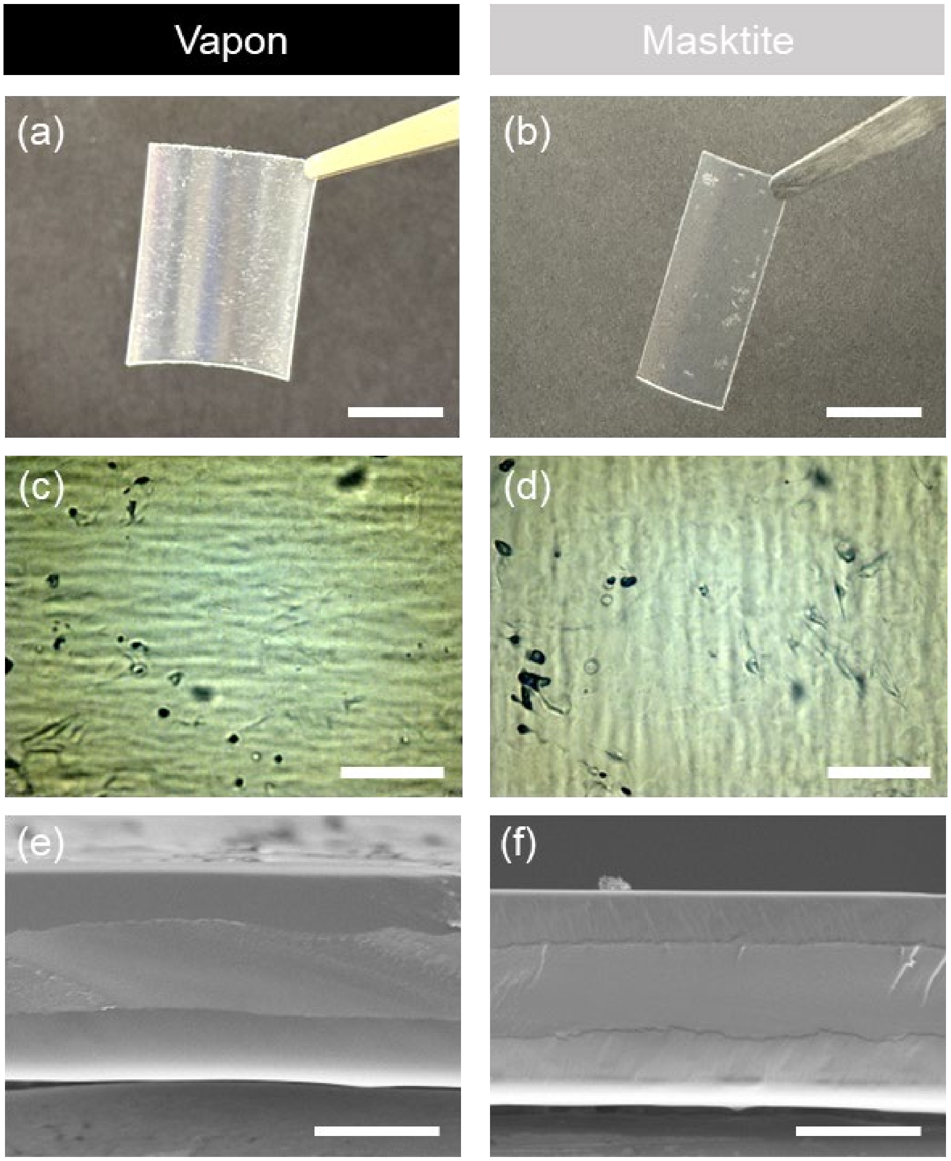

Figure S6. (a, b) Digital images of tapes, **(c, d)** Optical and **(e, f)** SEM micrographs of commercial medical-graded double-sided adhesives. Scale bars: (a-c) 10 mm, (d-f): 200 µm. (g-i): 50 µm.

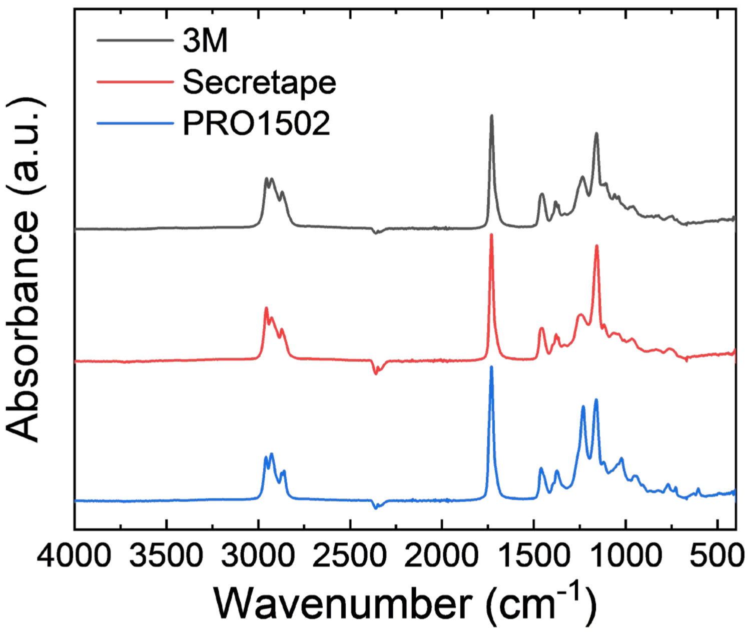

Figure S7. FT-IR spectroscopy of selected adhesive materials.

Characteristic peak assignments confirm modified acrylic and aliphatic composition:

2950 – 2960 cm^{-1} : asymmetric CH_3 stretching vibration[1,2]

2926 – 2928 cm^{-1} : CH_2 asymmetrical stretching[2,3]

2860 – 2870 cm^{-1} : symmetric CH_3 stretching vibration[2,3]

1730 cm^{-1} : C=O stretching vibration[1,2]

1455 – 1460 cm^{-1} : CH_2 asymmetric methyl (C-H) bending deformation mode[1,2]

1375 – 1380 cm-1 : symmetric methyl (C-H) bending deformation mode[2,3]

1230 – 1240 cm-1 : C-O stretching vibrations of the ester groups[1,2]

1160 cm-1 : C-O stretching vibrations of the ester groups[1,2]

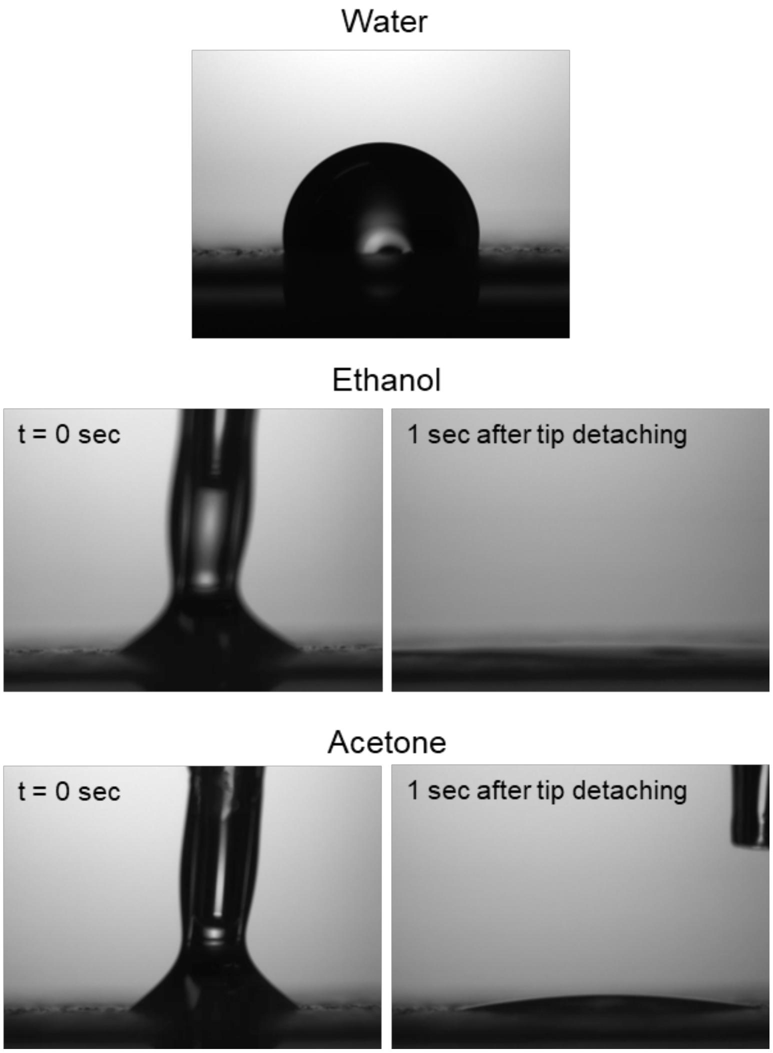

Figure S8. Contact angle measurements for ethanol and acetone on 3M PSA.

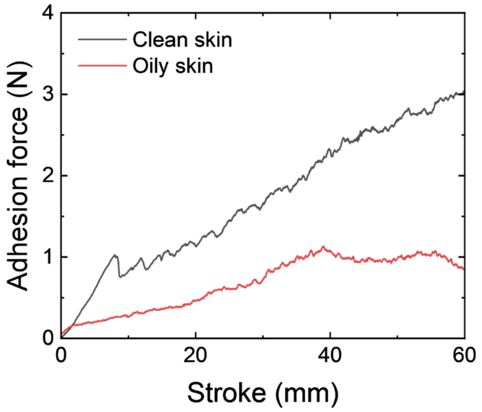

Figure S9. Pulling forces of 3M PSA tapes to separate from clean skin and oily skin.

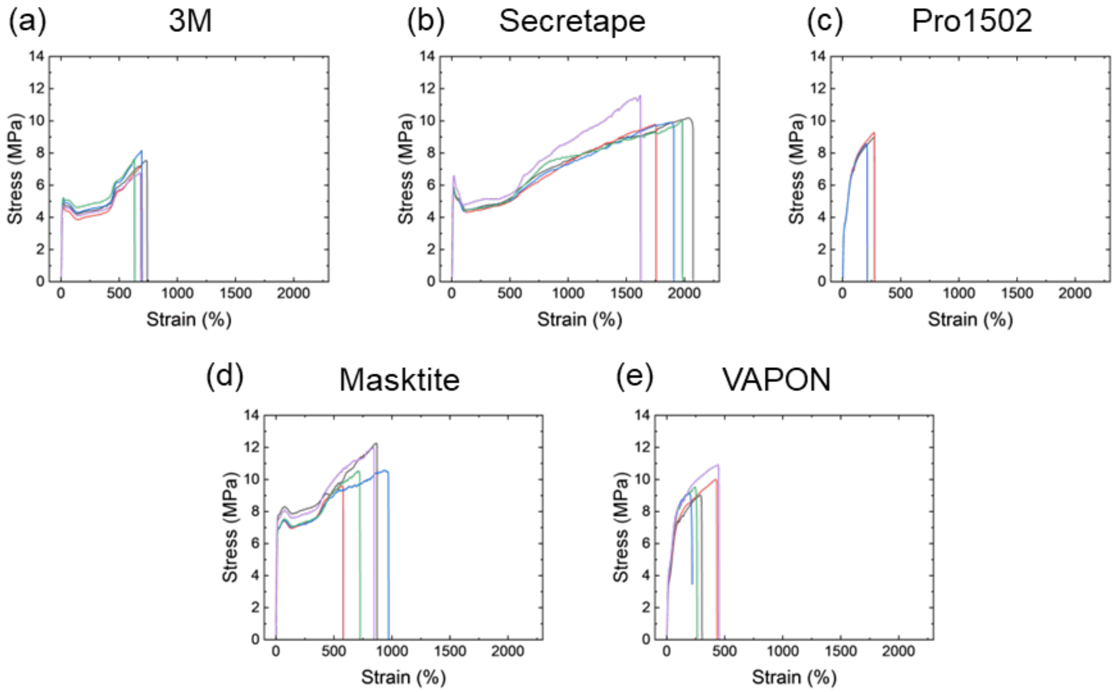

Figure S10. Stress strain curves of PSA films. (a) 3M, (b) Secretape, (c) Pro1502, (d) Masktite, and (e) VAPON.

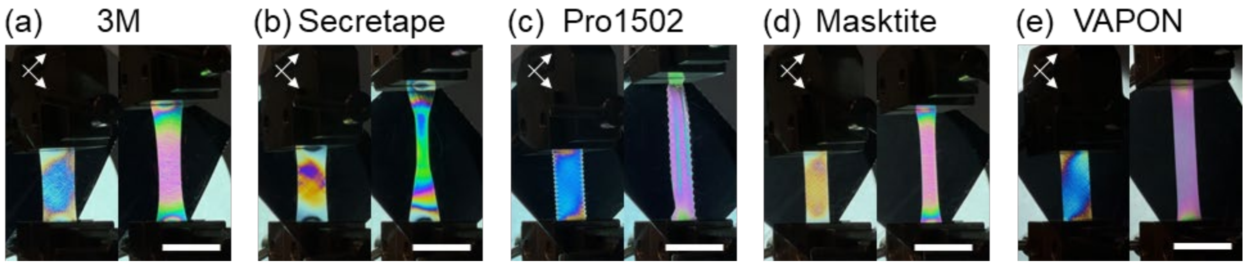

Figure S11. Crossed polarized photos of PSA tapes during different elongations. (left: 25%, right - a: 110%, b: 130%, c: 135%, d: 110%, and e: 135%) (a) 3M, (b) Secretape, (c) Pro1502, (d) Masktite, and (e) VAPON. Scale bars: 2 cm.

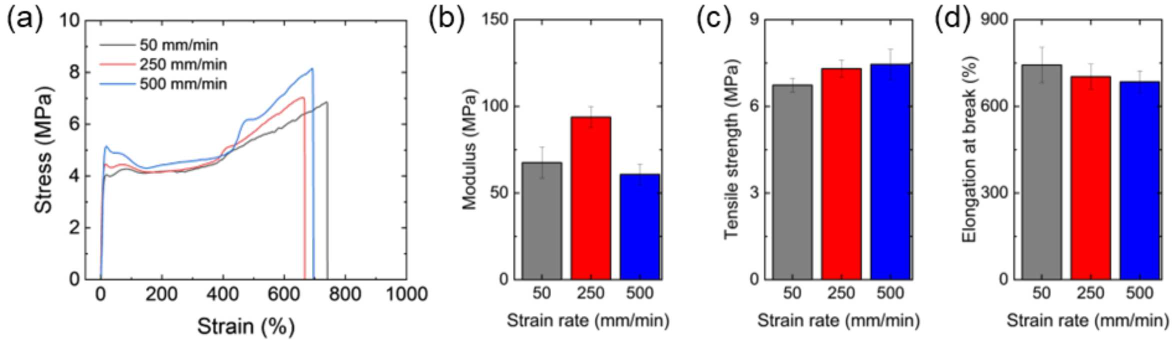

Figure S12. Mechanical properties of 3M PSA tape at different strain rates. (a) Stress-strain curve, (b) modulus, (d) elongation at break.

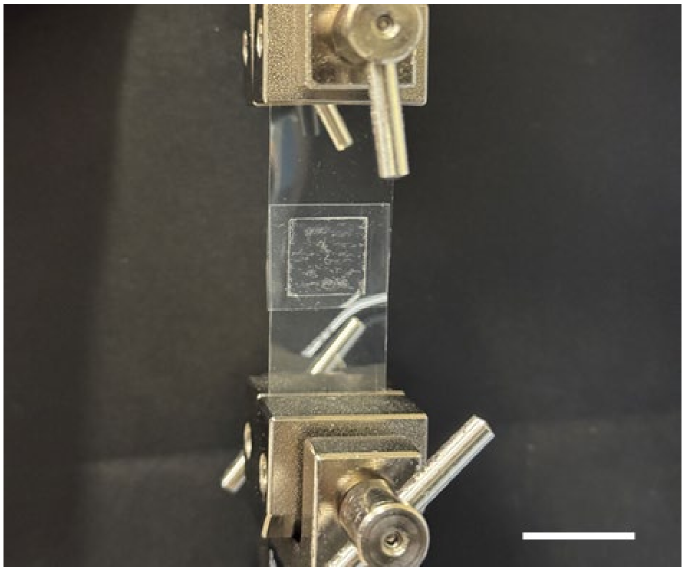

Figure S13. Set-up for lap shear tests of PSAs. Scale bar: 2 cm.

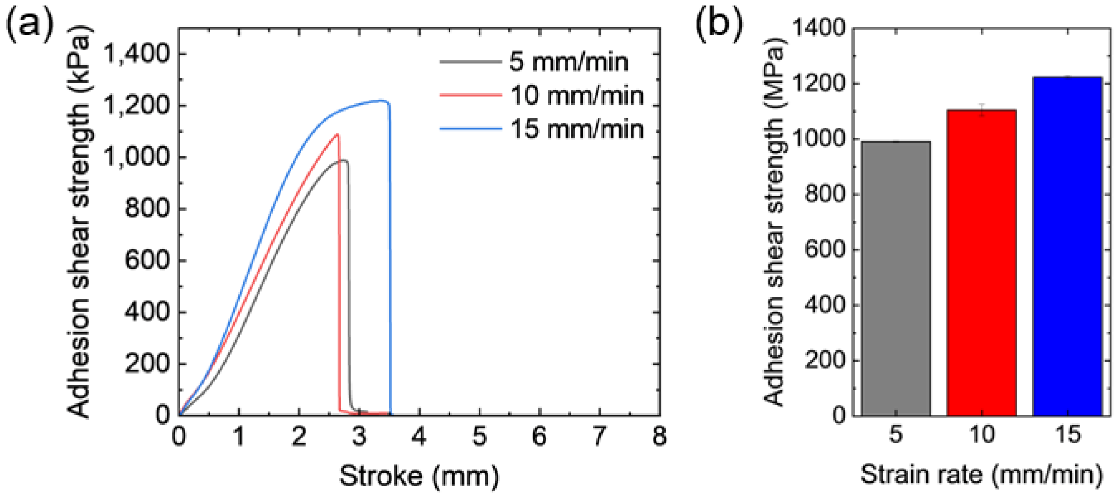

Figure S14. Lap shear test results of 3M PSAs at different shear rates. (a) Adhesion shear strength-stroke data of 3M PSA tapes. (b) Adhesion shear strength variation.

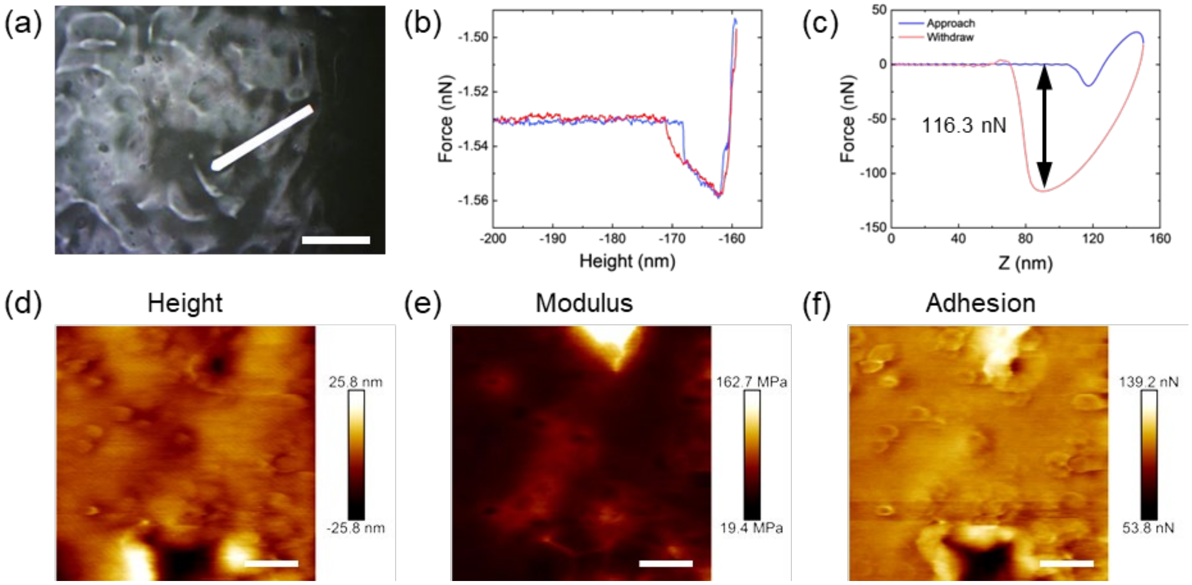

Figure S15. AFM QNM analysis of 3M PSA film. (**a**) Broken cantilever was caused by extremely high surface adhesion. Scale bar: 200 µm. Force distance data for: (**b**) non-adhesive glass substrate and (**c**) highly adhesive 3M PSA surface (pay attention to by two orders of magnitude higher force scale and four orders of magnitude difference in pull-off forces, 115 nN for PSA vs 0.03 nN for glass. QNM mapping of adhesive layers: (**d**) topography, (**e**) modulus, and (**f**) adhesion. Scale bars: 200 nm. Large indentation is visible on the bottom due to initial engagement under high attraction conditions.

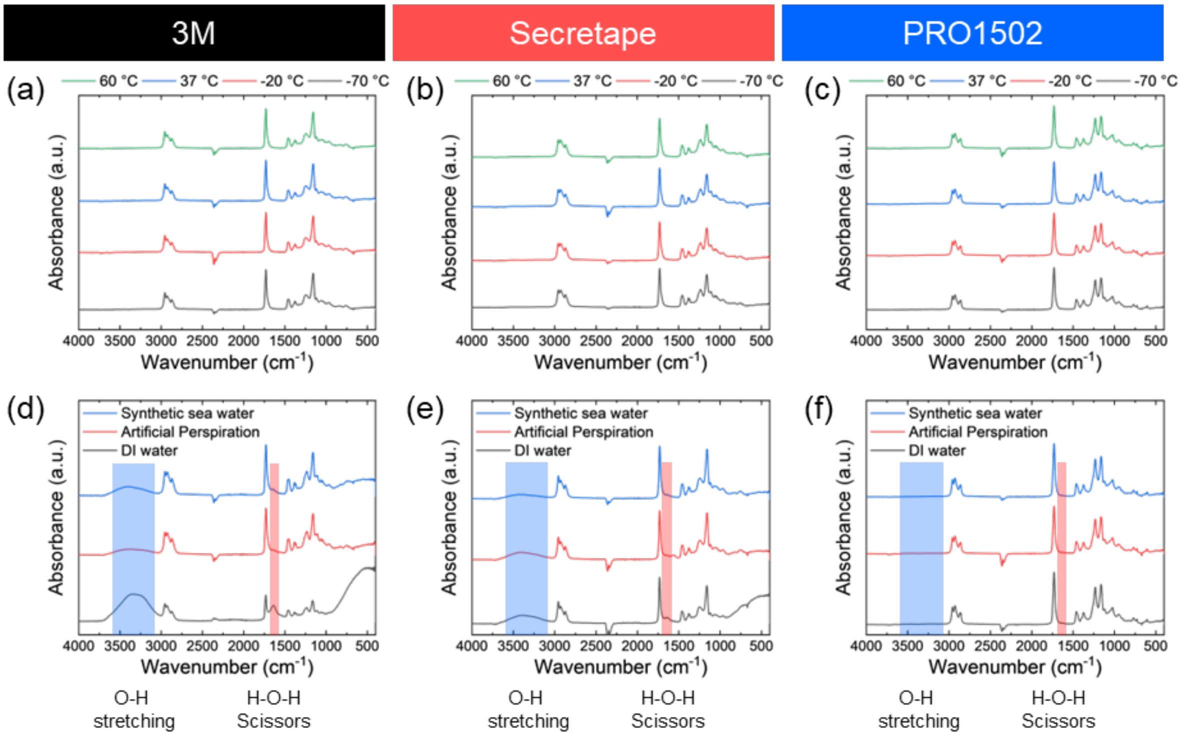

Figure S16. FT-IR spectroscopy of PSA films stored under different temperatures. (a, b, c) different temperatures, (d, e, f) water-based liquids.

The peaks after temperature treatments have negligible changes compared to the initial state of PSAs. This implies that the PSAs are chemically stable under human-centric extreme environments. The PSAs immersed under different water conditions, the new peaks correspond for FT-IR spectrum of water.

The main characteristic peak positions are:

Around 3300 cm^{-1}: hydroxyl bond (-OH) peak [3,4]

1640 cm^{-1}: H-O-H scissoring peak [2,4]

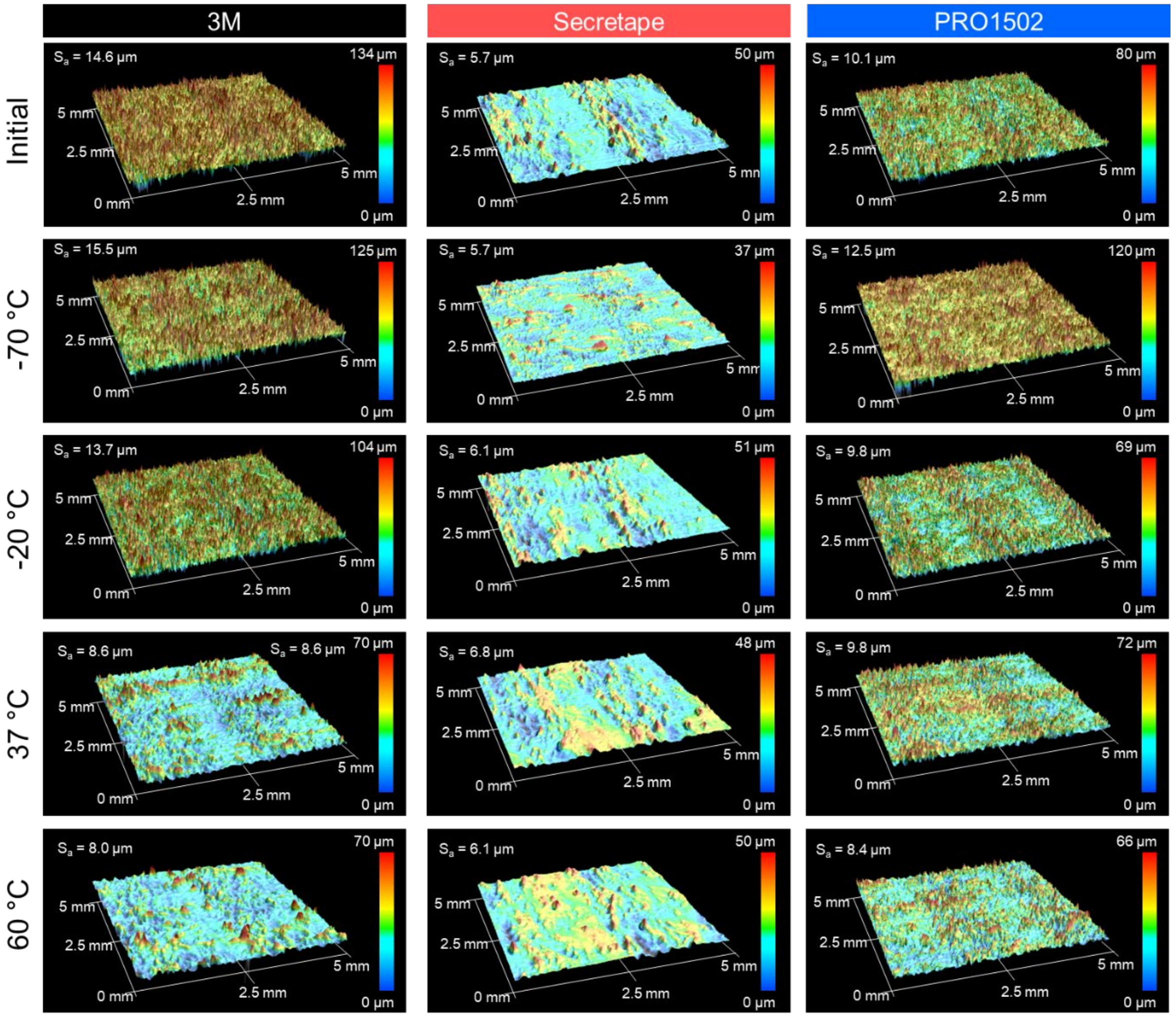

Figure S17. Surface profiles of all PSA films treated under various temperatures with measured surface roughness as left top insets in all panels.

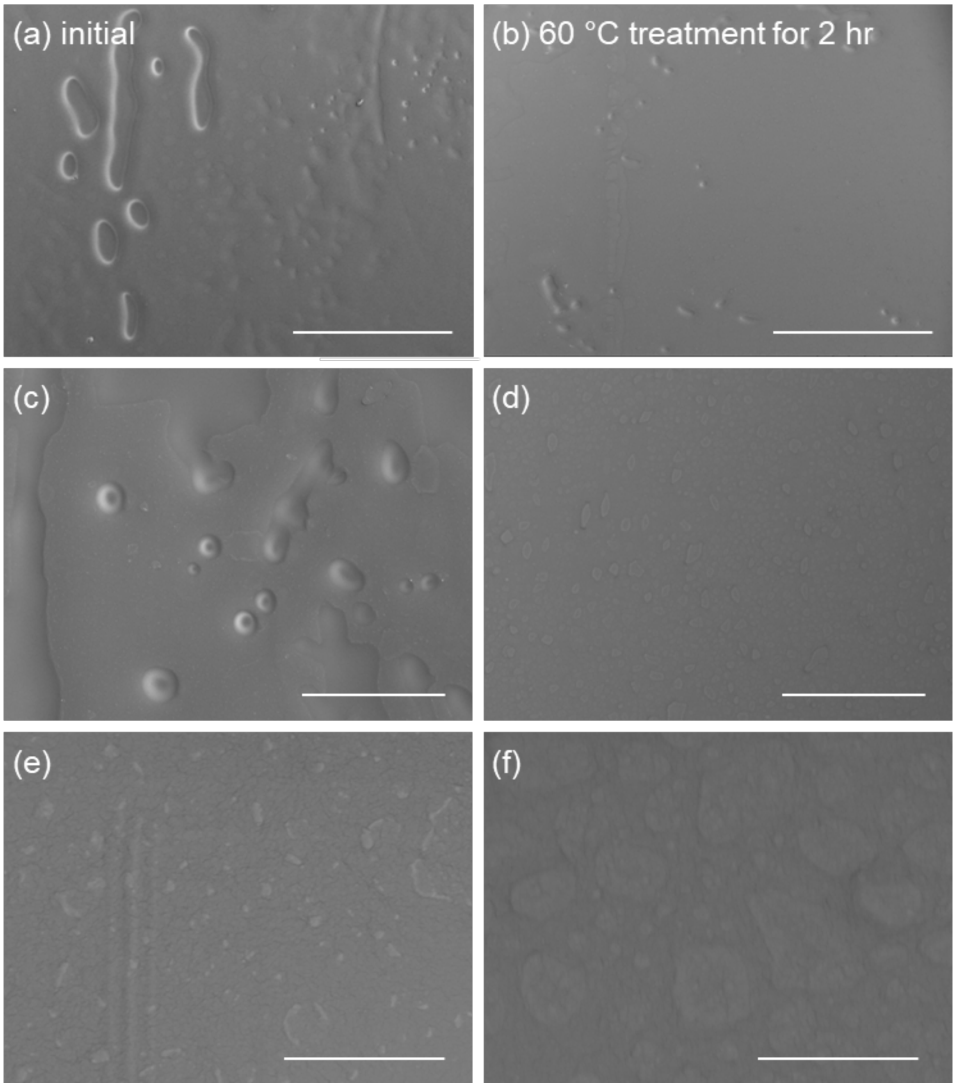

Figure S18. SEM micrographs of interfaces between glass substrate and 3M PSA before/after thermal treatments. Scale bars: (a, b) 1 mm, (c, d) 200 μm, (e, f) 20 μm.

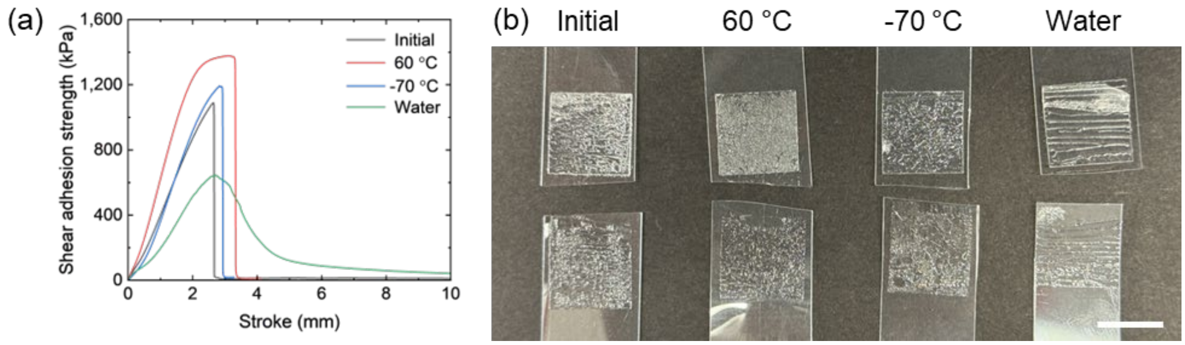

Figure S19. (a) Lap shear test results after storage under different environments. (b) Surface of pulled 3M PSA tapes separated from substrates, initial and those which have been stored under different environments for two weeks. Scale bar: 1 cm.

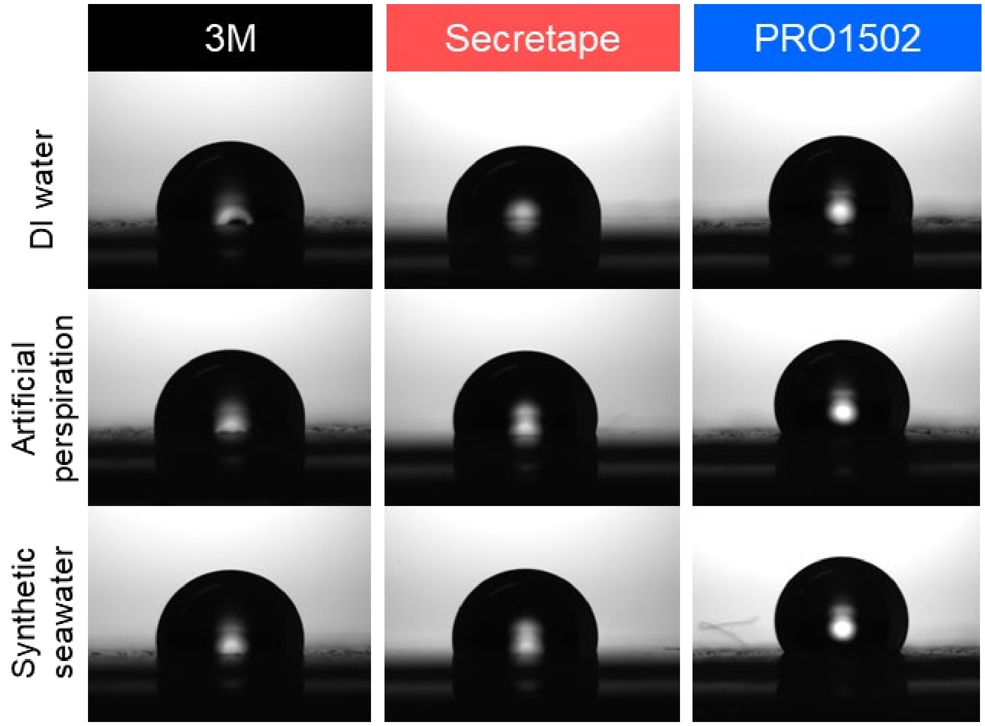

Figure S20. Contact angles of dried PSAs after different liquid treatments.

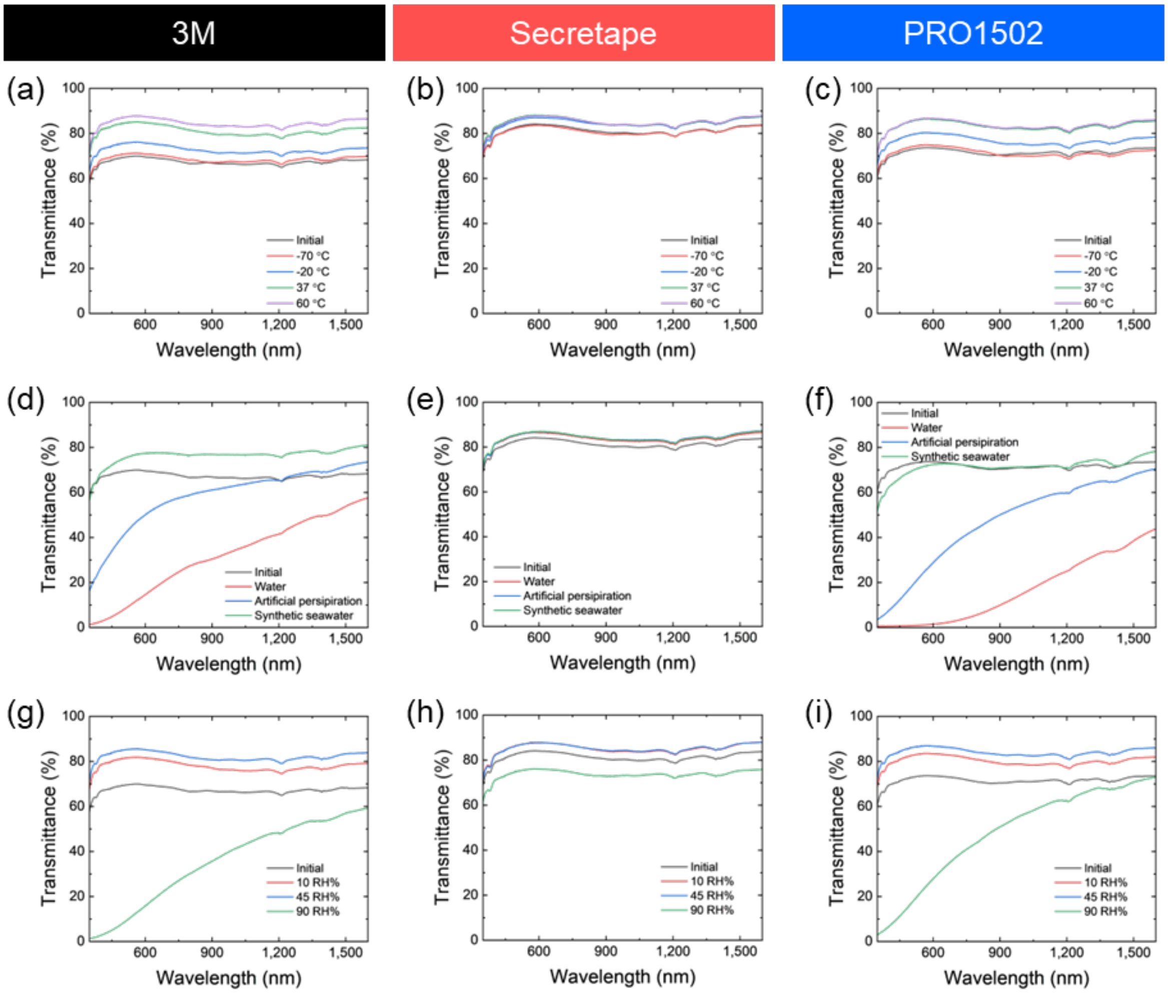

Figure S21. Optical transmittance of PSAs tapes under different conditions: (a, b, c) temperatures, (d, e, f) waters, (g, h, i) humidities.

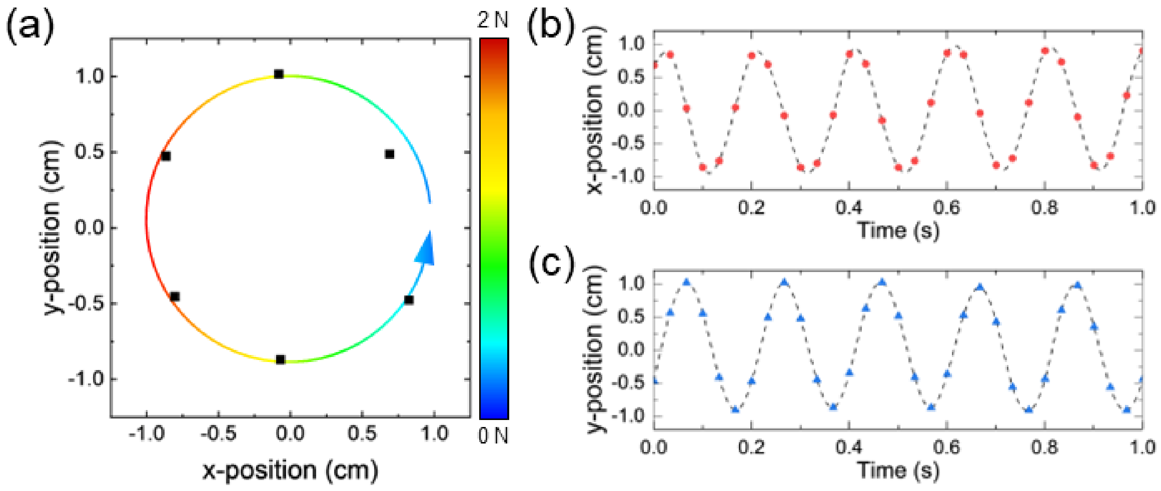

Figure S22. (**a**) Trajectory of circular vibrations with applied force for cyclical loading testing. (**b**) x- and (**c**) y-position of circular vibration loading.

Table S1. Maximum value of detachment force.

Average adhesive force	
- Without: 7.47 N<br>- Water: 6.75 N<br>- Ethanol: 0.94 N<br>- Acetone: 0.33 N	- Hand sanitizer: 4.3 N<br>- Acetone spraying: 0.47 N<br>- Adhesive remover: 0.37 N

Table S2. Surface roughness of film stored at different temperatures.

	3M	Secretape	PRO1502
Initial	14.6 μm	5.7 μm	10.1 μm
-70 °C	15.5 μm	5.7 μm	12.5 μm
-20 °C	13.7 μm	6.1 μm	9.8 μm
37 °C	8.6 μm	6.8 μm	9.8 μm
60 °C	8.0 μm	6.1 μm	8.4 μm

Supplementary References

[1] Kawasaki, A.; Furukawa, J.; Tsuruta, T.; Wasai, G.; Makimoto, T. Infrared Spectra of Poly(Butyl Acrylates). *Die Makromol. Chemie* **1961**, *49*, 76–111.

[2] Gorassini, A.; Adami, G.; Calvini, P.; Giacomello, A. ATR-FTIR Characterization of Old Pressure Sensitive Adhesive Tapes in Historic Papers. *J. Cult. Herit.* **2016**, *21*, 775–785.

[3] Huang, N.; Wang, J. A TGA-FTIR Study on the Effect of CaCO3 on the Thermal Degradation of EBA Copolymer. *J. Anal. Appl. Pyrolysis* **2009**, *84* (2), 124–130.

[4] Mojet, B. L.; Ebbesen, S. D.; Lefferts, L. Light at the Interface: The Potential of Attenuated Total Reflection Infrared Spectroscopy for Understanding Heterogeneous Catalysis in Water. *Chem. Soc. Rev.* **2010**, *39* (12), 4643–4655.
